# Virally encoded interleukin-6 facilitates KSHV replication in monocytes and induction of dysfunctional macrophages

**DOI:** 10.1371/journal.ppat.1011703

**Published:** 2023-10-26

**Authors:** Michiko Shimoda, Tomoki Inagaki, Ryan R. Davis, Alexander Merleev, Clifford G. Tepper, Emanual Maverakis, Yoshihiro Izumiya

**Affiliations:** 1 Department of Dermatology, School of Medicine, University of California, Davis, Sacramento, California, United States of America; 2 UC Davis Comprehensive Cancer Center, Sacramento, California, United States of America; 3 Department of Pathology and Laboratory Medicine, School of Medicine, University of California, Davis, Sacramento, California, United States of America; 4 Department of Biochemistry and Molecular Medicine, School of Medicine, UC Davis, Sacramento, California, United States of America; 5 Department of Biochemistry and Molecular Medicine, School of Medicine, University of California, Davis, Sacramento, California, United States of America; University of Southern California, UNITED STATES

## Abstract

Kaposi’s sarcoma-associated herpesvirus (KSHV) is an oncogenic double-stranded DNA virus and the etiologic agent of Kaposi’s sarcoma and hyperinflammatory lymphoproliferative disorders. Understanding the mechanism by which KSHV increases the infected cell population is crucial for curing KSHV-associated diseases. Using scRNA-seq, we demonstrate that KSHV preferentially infects CD14^+^ monocytes, sustains viral lytic replication through the viral interleukin-6 (vIL-6), which activates STAT1 and 3, and induces an inflammatory gene expression program. To study the role of vIL-6 in monocytes upon KSHV infection, we generated recombinant KSHV with premature stop codon (vIL-6(-)) and its revertant viruses (vIL-6(+)). Infection of the recombinant viruses shows that both vIL-6(+) and vIL-6(-) KSHV infection induced indistinguishable host anti-viral response with STAT1 and 3 activations in monocytes; however, vIL-6(+), but not vIL-6(-), KSHV infection promoted the proliferation and differentiation of KSHV-infected monocytes into macrophages. The macrophages derived from vIL-6(+) KSHV infection showed a distinct transcriptional profile of elevated IFN-pathway activation with immune suppression and were compromised in T-cell stimulation function compared to those from vIL-6(-) KSHV infection or uninfected control. Notably, a viral nuclear long noncoding RNA (PAN RNA), which is required for sustaining KSHV gene expression, was substantially reduced in infected primary monocytes upon vIL-6(-) KSHV infection. These results highlight the critical role of vIL-6 in sustaining KSHV transcription in primary monocytes. Our findings also imply a clever strategy in which KSHV utilizes vIL-6 to secure its viral pool by expanding infected monocytes via differentiating into longer-lived dysfunctional macrophages. This mechanism may facilitate KSHV to escape from host immune surveillance and to support a lifelong infection.

## Author summary

Kaposi’s sarcoma-associated virus (KSHV) is the causative agent of highly inflammatory diseases that include Kaposi’s sarcoma and KSHV-inflammatory cytokine syndrome (KICS). Macrophages are important immune cells that regulate inflammation by stimulating both innate and adaptive immune systems. A small fraction of monocytes differentiates into macrophages to acquire a longer life span and migrate to residential areas. Deregulation of macrophage functions weakens host immune defense mechanisms, allowing secondary infections resulting in prolonged inflammation. Here, we demonstrate that KSHV infection to monocytes facilitates their transition into macrophages; however, those infected macrophages have impaired immune stimulatory function. Such tradition depends on the expression of KSHV vIL-6, a virally encoded IL-6 homolog. Prolonged vIL-6 stimulation in culture also induced a similar phenotype in monocytes. These results suggest that continuous stimulation by KSHV-derived vIL-6 deregulates host macrophage functions. This mechanism may be associated with hyperinflammatory phenotypes seen in KSHV-associated diseases.

## Introduction

A virus is an infectious agent that can only replicate within a living host organism. Because of this dependence, viruses have evolved mechanisms to exploit normal cell functions to escape host immune surveillance for their survival advantage. This exploitation is sometimes associated with prolonged damage to the host, leading to pathologic processes and diseases caused by the viral infection [1].

Kaposi’s sarcoma (KS)-associated herpesvirus (KSHV), or human gamma herpesvirus 8 (HHV-8), is an oncogenic double-stranded DNA virus that establishes a lifelong latent infection [2]. KSHV is the etiologic agent of Kaposi’s sarcoma and is associated with two lymphoproliferative disorders: multicentric Castleman’s disease (MCD) and HIV-associated primary effusion lymphoma (PEL). KSHV-inflammatory cytokine syndrome (KICS) may also represent a prodromic form of KSHV-MCD, which exhibits elevated KSHV viral loads and circulating inflammatory cytokines including IL-6, IL-10, and a KSHV-encoded IL-6 homolog (vIL-6) [3–8]. These highly inflammatory diseases are devastating and a leading cause of cancer deaths in people living with HIV. Therefore, understanding the mechanism of KSHV infection and its association with inflammatory disease development is crucial for finding a cure for these diseases.

Natural transmission of KSHV most likely occurs through salivary and sexual transmission or during transplantation of KSHV-positive organs into a naïve recipient, although initial KSHV infection is typically asymptomatic [2,9]. In experimental settings, KSHV has been shown to infect various types of cell lines and primary cells, such as epithelial cells and immune cells that include B cells, monocytes, and dendritic cells through binding to specific cell surface receptors such as Siglec DC-SIGN [10–14]. However, it remains unclear as to whether KSHV may strategically infect a particular cell type among PBMC. The mechanisms by which KSHV facilitates a lifelong infection by increasing viral reservoirs and impacts the host immune system are also not entirely clear.

KSHV-encoded viral interleukin-6 (vIL-6) is a homolog of human interleukin-6, which is encoded by KSHV ORF-K2 and is highly expressed during the lytic replication cycle [15]. Viral IL-6 is also expressed at physiologically functional levels in latently infected cells [16] and is detectable in the sera and/or tumor tissues of patients with KS, PEL, and MCD [17]. Viral IL-6 enhances cell proliferation, endothelial cell migration, and angiogenesis, leading to tumorigenesis, and has been suggested to be a driver of KICS [18,19]. In addition, vIL-6 transgenic mice developed IL-6-dependent MCD-like disease [20] and supported tumor metastasis in a murine xenograft model [21]. Mechanistically, vIL-6 directly binds to the gp130 subunit of the IL-6 receptor without the need for the IL-6 receptor α, and actives the JAK/STAT pathway to induce STAT3 phosphorylation and acetylation [8,22,23]. In addition, vIL-6 activates the AKT pathway to promote numerous oncogenic phenotypes [18,19,24,25]. STAT3 activation by vIL-6 also increases the VEGF expression through the downregulating caveolin 1 [8] and promotes angiogenesis, suggesting that vIL-6 plays an important role in tumorigenesis through STAT activation. Given that the prototypical human IL-6 plays a critical role in immune regulation and inflammation, vIL-6 is thought to play a pivotal role in inflammatory KSHV diseases. Here, we reveal the role of vIL-6 in the regulation of monocytes by utilizing recombinant KSHV and *de novo* infection to the peripheral blood mononuclear cells.

## Results and discussion

### KSHV preferentially infects CD14^+^ monocytes among PBMC, triggering an inflammatory response and macrophage differentiation

To study cell type-specific KSHV infection, we employed a single cell (sc)RNA-seq analysis approach. Recombinant KSHV (rKSHV.219) virions were purified by two serial ultracentrifugations from the culture supernatant of the iSLK.219 cell line, an inducible recombinant KSHV producer cell. Peripheral blood mononuclear cells (PBMCs) were infected with rKSHV.219 at MOI = 1, fixed at various time points (day 0, 1, 2, and 4) after infection, and subjected to scRNA-seq analysis. KSHV infection and lytic replication in single cells were then monitored by the expression of all KSHV genes.

**Fig 1:**
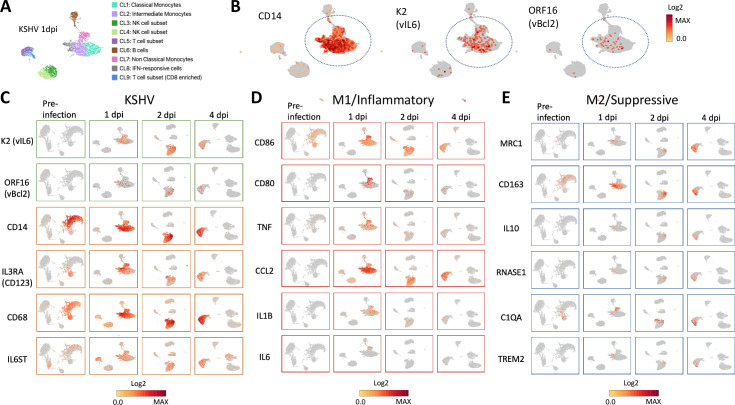
KSHV preferentially infects monocytes and triggers the monocyte inflammatory transcription program. PBMCs were infected with rKSV.219 at MOI = 1, fixed at various time points (pre-infection, day 1, 2, and 4) after infection, and subjected to 10x Genomics Chromium scRNA-seq analysis. (A) UMAP visualizes 9 PBMC clusters (CL1: Classical Monocytes, CL2: Intermediate Monocytes, CL3 and CL4: NK cell subset, CL5: T cell subset, CL6: B cells, CL7: Non Classical Monocytes, CL8: IFN-responsive cells, and CL9: CD8 enriched T cell subset) in a 1 day-post-infection (dpi) sample based on lineage-specific gene expression of *KLRD1*, *CD14*, *APOBEC3A*, *VMO1*, *CD79A*, and *CD3E* genes for NK cells, monocytes, intermediate monocytes, non-classical monocytes, B cells, and T cells, respectively. The detailed list of differentially expressed genes for each cluster is shown in S1 Table. (B) KSHV-encoded K2 (vIL-6) and ORF16 (vBCL2) expression overlapped with CD14 expression at 1 dpi. (C) The kinetic scRNA-seq analysis on pre-infection, 1 dpi, 2 dpi, and 4 dpi revealed that the colocalized expression of KSHV-encoded K2 and ORF16 and the monocyte-related genes *CD14*, *IL3RA (CD123)*, *CD68*, *and IL6ST (gp130)*. (D) KSHV infection in CD14^+^ monocytes triggered the activation and inflammatory response at 1 dpi with the upregulation of *CD86*, *CD80*, *TNFα*, *CCL2*, *IL1b*, and *IL6*. (E) KSHV infection in CD14^+^ monocytes later upregulated the expression of genes associated with differentiation of M2-like macrophages with suppressive phenotype (*MRC1*, *CD163*, *IL10*, *RNASE1*, *C1QA*, *and TREM2*). Gene expression levels are indicated by heatmap bars. A representative of three similar experiments is shown.

As shown in Fig 1A, unsupervised Uniform Manifold Approximation and Projection (UMAP) for dimension reduction analysis identified 9 clusters among peripheral blood mononuclear cells (PBMCs). The detailed list of differentially expressed genes for each cluster is shown in S1 Table. Each of the clusters was then associated with known immune cell subsets based on corresponding lineage-specific gene expression. Thus, *KLRD1(CD94)*, *CD14*, *APOBEC3A*, *VMO1*, *CD79A*, and *CD3E* genes were used as guiding markers for the classification of NK cells, monocytes, intermediate monocytes, non-classical monocytes, B cells, and T cells, respectively, based on the immune cell data available from the Human Protein Atlas [26] (S1A Fig). All four types of immune cells were present during 4-day infection period, and monocyte cell population was slightly decreased at day 4. (S1B Fig).

As shown in Fig 1B, *de novo* KSHV infection of PBMCs in conjunction with scRNA-seq analysis at 1-day post-infection (dpi) revealed that K2 (vIL-6) and ORF16 (vBCL2) had the two most sequence reads detected among all the KSHV open reading frames (S2A Fig). These KSHV reads were almost entirely overlapped with CD14 expression. It should be noted that scant K2 expression was also found in *CD79A*^+^ B cells, *KLRD1*^+^ NK, and *CD3E*^+^ T cell clusters (Figs 1B and S2). KSHV has been shown to establish latent infection in B cells, causing B-cell lymphoma [2]. However, the correlation in expression of CD14 and K2 was highly significant (p = 8 x 10^−191^) compared to that of *CD19* and K2 (p = 0.065) at 1 dpi. The following kinetic scRNA-seq analysis showed that the expression of both K2 and ORF16 and the host gene expression associated with monocytes such as *CD14, IL3RA(CD123)*, and *CD68*, as well as *IL6ST* (gp130), the receptor of vIL-6 [8,22,23], was colocalized during 1 to 4 dpi (Fig 1C). Using eGFP reporter gene expression under human EF-1 promoter in rKSHV.219-infected cells, we confirmed that eGFP-positive cells mainly express CD14, HLA-DR, and CD11c but not lineage markers for B cells (CD19) or T cells (CD3), and that ∼70% of CD14^+^ cells were eGFP-positive (S3 Fig). By intracellular staining of vIL-6 at 2 dpi, we also confirmed that the majority (>80%) of CD14^+^ monocytes expressed high levels of vIL-6 protein whereas ∼50% of non-CD14^+^ lymphocytes were weakly stained with antibody against vIL-6 (S4 Fig). In this experiment, the culture medium was not replenished during the period. Infectious KSHV virions that remained in the culture or were newly released from infected cells could have infected other cell subsets of PBMC besides CD14^+^ monocytes. Based on these results, we concluded that KSHV preferentially infects CD14^+^ monocytes and that monocytes can support KSHV lytic replication along with vIL-6 protein expression.

The kinetic scRNA-seq analysis also revealed that KSHV infection in CD14^+^ monocytes triggered the activation and inflammatory response immediately after KSHV infection at 1 dpi as evidenced by the upregulation of *CD86*, *CD80*, *TNFα*, *CCL2*, *IL1β*, and *IL6* (Fig 1D). The latter was followed by the expression of genes associated with differentiation of M2-like and Tumor-associated macrophages with a suppressive phenotype (*MRC1*, *CD163*, *IL10*, *RNASE1*, *C1QA*, and *TREM2*) [27–29] during 2–4 dpi (Fig 1E). Collectively, scRNA-seq analysis demonstrates that KSHV infection triggers a monocyte inflammatory response followed by a macrophage differentiation program.

### KSHV infection of PBMC triggers STAT activation in dendritic cells and myeloid cell lineages

As a counterpart of the pleiotropic cytokine human IL-6, viral IL-6 has been shown to exhibit multifunctional pathologic roles in KS diseases [15]. Therefore, we next studied the role of vIL-6 in monocyte activation and differentiation using a *de novo* PBMC infection model. To this end, we generated recombinant KSHV lacking vIL-6 expression by inserting stop codons in vIL-6 (vIL-6STOP) using the KSHV BAC clone BAC16 (S5 Fig). We also generated a revertant KSHV with intact vIL-6 expression (vIL-6REV) by changing the sequence back to the original wild-type sequence with BAC recombination (S5A–S5C Fig). The lack of vIL-6 expression in vIL-6STOP infected iSLK cells was confirmed by Western blotting using a monoclonal antibody against vIL-6 (a kind gift from Dr. Robert Yarchoan, NIH) in reactivated iSLK cells (S5D Fig). We also confirmed that wild type BAC16 and vIL-6REV showed equivalent levels of lytic gene induction in iSLK cells with Doxycycline and TPA treatment as evident by similar levels of K-Rta and K-bZIP protein expression (S5D Fig). We also measured virion production in culture supernatant (S5E Fig), which showed a wild-type level of virions were produced by vIL-6REV-infected iSLK cells. These results validate that vIL-6REV has equivalent functionality to the wild-type BAC16. Consistent with previous report, vIL-6STOP showed reduced viral gene transcription and encapsurated KSHV virions [30].

To evaluate the signaling events triggered by KSHV infection in immune cells, PBMC from healthy donors (n = 6, 3 males and 3 females) were infected with vIL-6REV (with vIL-6 expression) or vIL-6STOP (MOI = 1), or mock-infected (PBS). At 1 dpi, cells were fixed and subjected to CyTOF analysis using a 27-color signaling panel that can differentiate 25 immune cell subsets (S6 Fig). The expression levels of 10 signaling molecules were then determined to evaluate the early signaling events in PBMCs upon KSHV infection.

**Fig 2:**
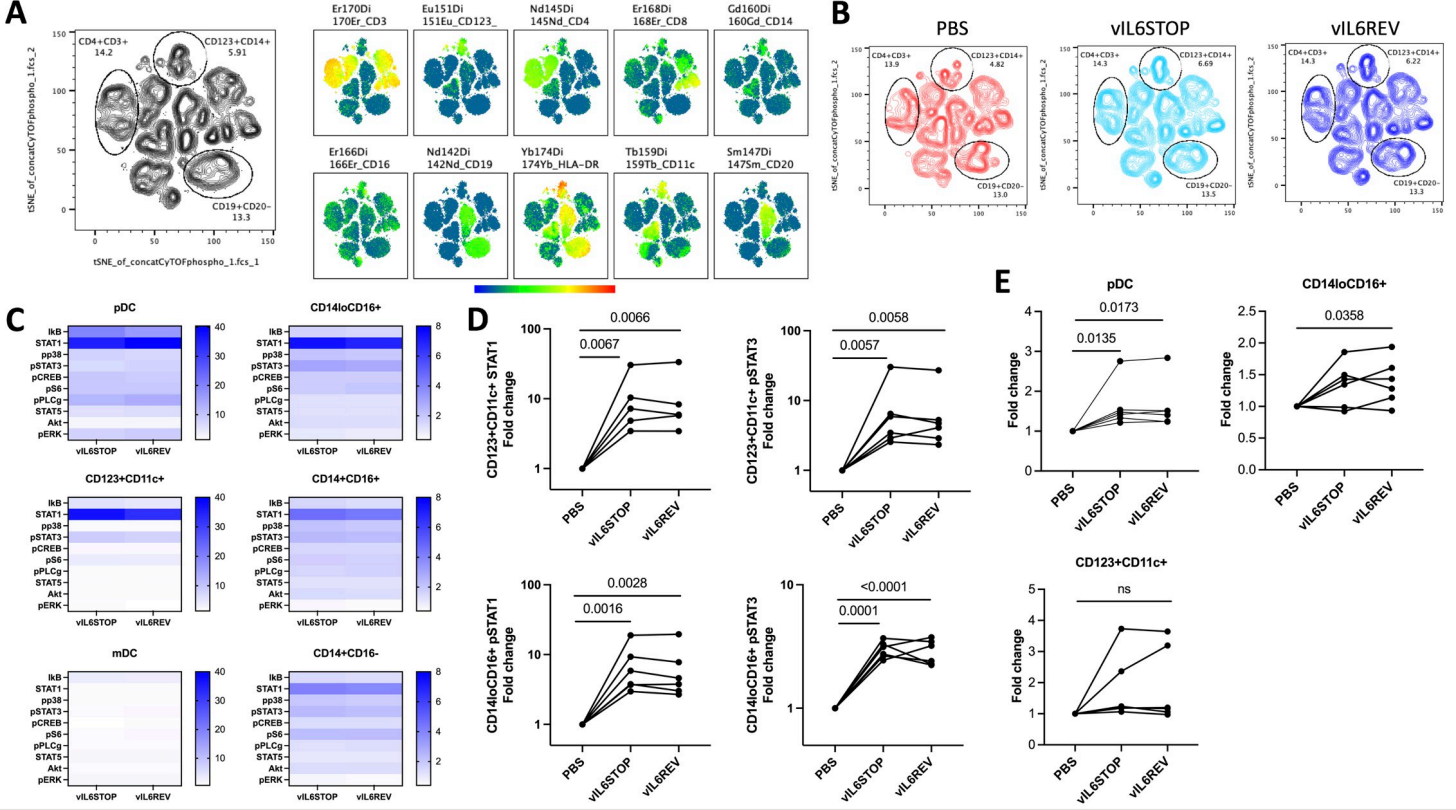
KSHV infection preferentially triggers STAT activation in monocytes and dendritic cells. PBMC from healthy donors (n = 6) were infected with vIL-6REV (KSHV with vIL-6 expression) or vIL-6STOP (KSHV without vIL-6 expression) (MOI = 1), or mock-infected (PBS). At 1 dpi, cells were fixed and subjected to CyTOF analysis. The expression levels of 10 signaling molecules were evaluated. Data combine two experiments (n = 3 each). (A) tSNE for dimension reduction analysis was applied for each infection group after 1000 events from 6 samples in each group were concatenated. Immune cell subsets were clustered based on each lineage’s marker expression (Right panel). (B) The phenotypic changes induced in three immune cell populations corresponding to CD4^+^CD3^+^, CD19^+^CD20^-^, and CD123^+^CD14^+^ clusters by infection were identified by comparison between vIL-6STOP, vIL-6REV, and the mock-infected (PBS) control group. (C) The activation status of 10 signaling molecules among plasmacytoid dendritic cells (pDC) and monocytic cell subsets, including CD123^+^CD11c^+^ cells, myeloid (m)DC, CD14^lo^CD16^+^ non-classical monocytes, CD14^+^CD16^+^ intermediate monocytes, and CD14^+^CD16^-^ classical monocytes are shown in heat maps. Each subset was identified based on the lineage cell surface marker expression with gating strategies shown in S6 Fig. (D) The fold change in the CyTOF signal intensity of STAT1 and pSTAT3 for CD123^+^CD11c^+^ cells (TOP panel) and CD14^+^CD16^+^ cells (Bottom panel) in infected groups compared to mock-infected (PBS) control group are shown. p values shown are by ratio paired t-test. p<0.05: statistically significant. (E) Individual percentages of pDCs, CD14^+^CD16^+^, and CD123^+^CD11c^+^ cells among DC gate for each PBMC sample (n = 6) are compared between vIL-6REV- and vIL-6STOP- infected groups, and the uninfected group (PBS). p values shown are by ratio paired t-test. p<0.05: statistically significant.

Unsupervised t-distributed stochastic neighbor embedding (tSNE) for dimension reduction analysis was applied for each infection group. As shown in Fig 2A, immune cell subsets were clustered based on each marker expression, and the phenotypic changes induced by infection were visualized and identified by comparison between vIL-6STOP, vIL-6REV, and the mock-infected (PBS) control group (Fig 2B). The landscape of immune cell phenotypes visualized by the tSNE cell clustering was remarkably similar between the vIL-6STOP and vIL-6REV infection group, indicating that the anti-viral response was equally triggered in monocytes in vIL-6STOP and vIL-6-REV infection. Nonetheless, the tSNE visualization revealed that the phenotype of three immune cell populations corresponding to CD4^+^CD3^+^, CD19^+^CD20^-^, and CD123^+^CD14^+^ clusters, as indicated in Fig 2B, was different between the infected groups and the mock-infected control group. The CD123^+^CD14^+^ cluster contains a heterogenous cellular population that also expresses CD11c, CD16, and HLA-DR (Fig 2A). Given that the scRNA-seq results showed preferential KSHV infection in the CD14^+^ cell population in association with CD123 (IL3R) upregulation (Fig 1C), we considered that the changes observed in the CD123^+^CD14^+^ cluster represent the host cell anti-virus response in monocytes after KSHV infection. Changes in CD4^+^CD3^+^ and CD19^+^CD20^-^ clusters could be the result of infection and/or indirect activation, such as through cytokine production from PBMCs in response to KSHV infection.

To evaluate signaling events in detail, we next used a knowledge-based analysis and identified 25 immune cell subsets based on the gating described in S6 Fig. The activation status of each signaling molecule was evaluated among plasmacytoid dendritic cells (pDC) and monocytic cell subsets, including CD123^+^CD11c^+^ cells, myeloid (m)DC, CD14^lo^CD16^+^ non-classical monocytes, CD14^+^CD16^+^ intermediate monocytes, and CD14^+^CD16^-^ classical monocytes, based on the fold change in the CyTOF signal intensity for infected groups compared to the mock-infected (PBS) control group (Fig 2C and 2D). As expected, many of the 10 signaling molecules, including STAT1, IkB, and phospho-PLCγ, were strongly upregulated in pDC (Fig 2C), an immune cell subset responsible for the anti-viral response. Among CD11c^+^ DC subsets, STAT1 and phospho-STAT3 were the two most significantly activated pathways in CD123^+^CD11c^+^ cells and less so in myeloid (m)DC (Fig 2C and 2D). Among monocyte subsets, STAT1, phospho-STAT3, and pS6 were upregulated after infection (Fig 2C and 2D). Consistent with the tSNE results, the activation profiles of 10 signaling molecules in pDC, DC, and monocytes were identical between vIL-6STOP and vIL-6REV infection (Fig 2C and 2D). Thus, at 1 dpi, both vIL-6STOP and vIL-6REV viruses triggered indistinguishable host responses in pDC, DCs, and monocytes.

In association with the upregulation of STAT1 by infection (Fig 2C and 2D), the frequency of the pDCs among dendritic cells at 1 dpi increased regardless of the vIL-6 expression (Fig 2E), while the change in the frequency for CD123^+^CD11c^+^ cells did not meet statistical significance. However, the frequency of the CD14^+^CD16^+^ population increased by infection, and the presence of vIL-6 expression seems to further enhance the activation of infected CD14^+^CD16^+^ monocytes (Fig 2E).

### KSHV infection promotes activation and proliferation of CD14^+^ monocytes in a manner dependent on vIL-6

**Fig 3:**
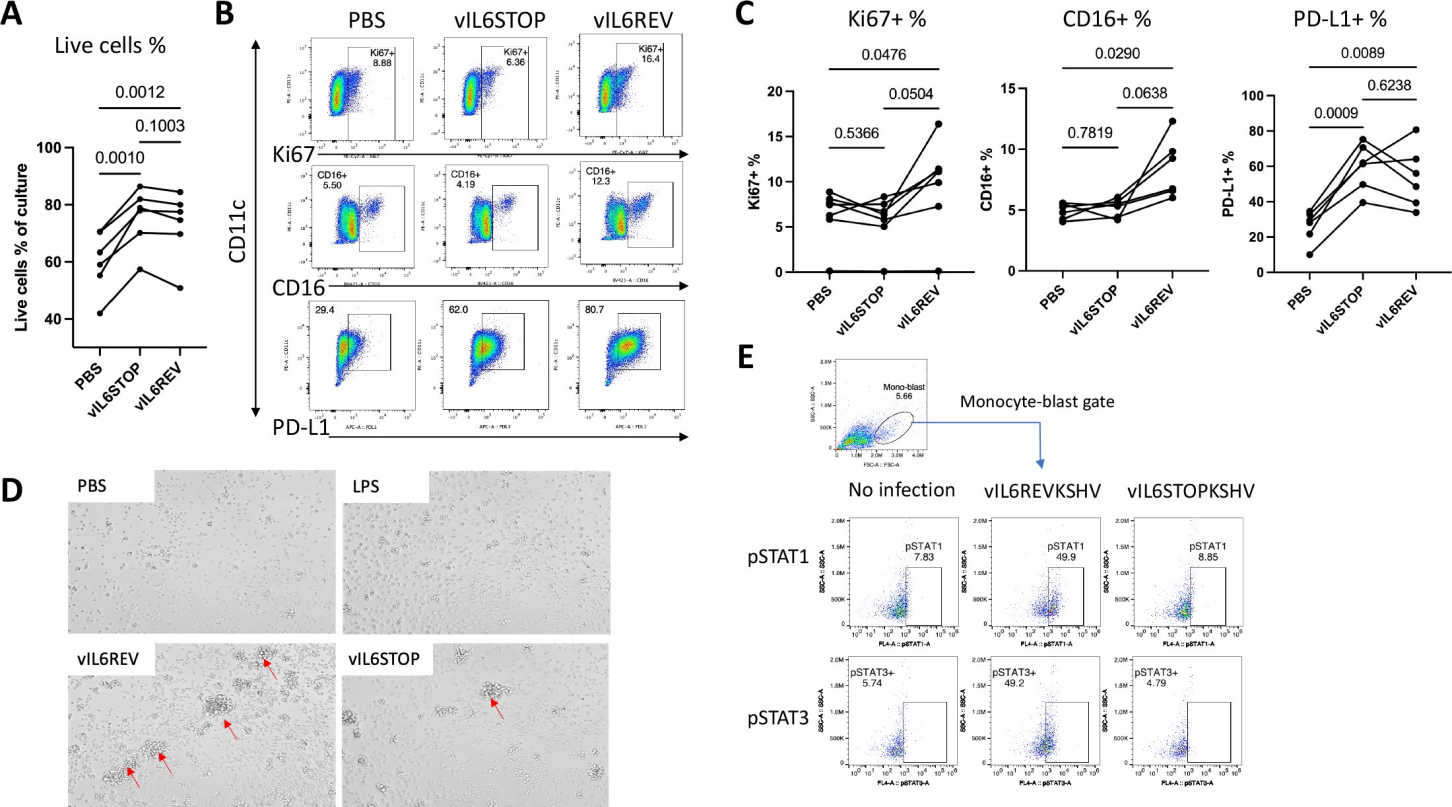
KSHV preferentially infects and expands CD14+ monocytes with an inflammatory response in a manner dependent on vIL-6. Monocytes were isolated negatively from PBMCs (n = 6) using magnetic beads-based negative enrichment and infected with vIL-6STOP or vIL-6REV rKSHV (MOI = 1). (A) The frequencies of live cells at 2 dpi by live/dead staining are shown. (B) Representative FACS profiles of infected monocytes analyzed by flow cytometry for Ki67, CD16, and PD-L1 expressing cells. (C) The frequencies for Ki67+, CD16+, and PD-L1+ cells are shown. Data represent two similar experiments. (D) Representative images of cell blasts in the indicated treatment conditions (x100). (E) Representative FACS profiles of each sample for pSTAT1 and pSTAT3 intracellular staining with the percentage of gated pSTAT1- and pSTAT3- expressing cells are shown. Data represent two similar experiments. *p* values shown are by ANOVA with a paired comparison with Tukey’s multiple comparison test. *p* < 0.05: statistically significant.

To further study the biological significance of vIL-6 expression and STAT 1 and 3 activations, monocytes were isolated from PBMCs (n = 6) using a magnetic beads-based negative enrichment method and infected with vIL-6STOP or vIL-6REV KSHV (MOI = 1). The viability and phenotype were then analyzed by flow cytometry at 2 dpi. KSHV infection increases CD274 (PD-L1) expression in human monocytes [31]. We found that total cell viability (Fig 3A) and the frequency of activated PD-L1^+^ cells (Fig 3B and 3C) were significantly increased by KSHV infection regardless of the expression of vIL-6. These results were expected based on the CyTOF signaling analysis results that demonstrate that the initial host response against vIL-6REV and vIL-6STOP infection was indistinguishable. The results were also supported by the observation that KSHV infection promptly induced inflammatory cytokine expression in monocytes (Fig 1D). Inflammatory cytokines such as TNFα produced by activated monocytes can support their survival in an autocrine manner during the inflammatory response [32]. Therefore, it is possible that the effect of vIL-6 on cell survival, if any, could be overridden by the effect of other inflammatory cytokines during the early host anti-viral response against KSHV.

On the other hand, the frequency of Ki67^+^ proliferating cells and that of CD16^+^ cells were significantly increased in a manner dependent on the presence of vIL-6 (Fig 3B and 3C). Consistent with flow cytometry analyses, larger and more frequent proliferating blast cells were observed in response to vIL-6REV infection compared to that of vIL-6STOP infection (Fig 3D). Intracellular staining of monocytes also confirmed that the frequency of pSTAT1 and pSTAT3 in monocytes continued to be higher in vIL-6REV-infection compared to that in vIL-6STOP or mock-infection at 2 dpi (Fig 3E), suggesting that vIL-6 expression is required for sustaining STAT activation. These results collectively suggest that vIL-6 expression during the early KSHV infection increases the proliferation of infected monocytes via STAT 1 and 3 activation. The cellular environment in monocytes may have a unique role in sustaining STAT activation with KSHV infection.

### KSHV infection changes the transcriptional landscape of macrophages in a manner dependent on vIL-6 expression

**Fig 4:**
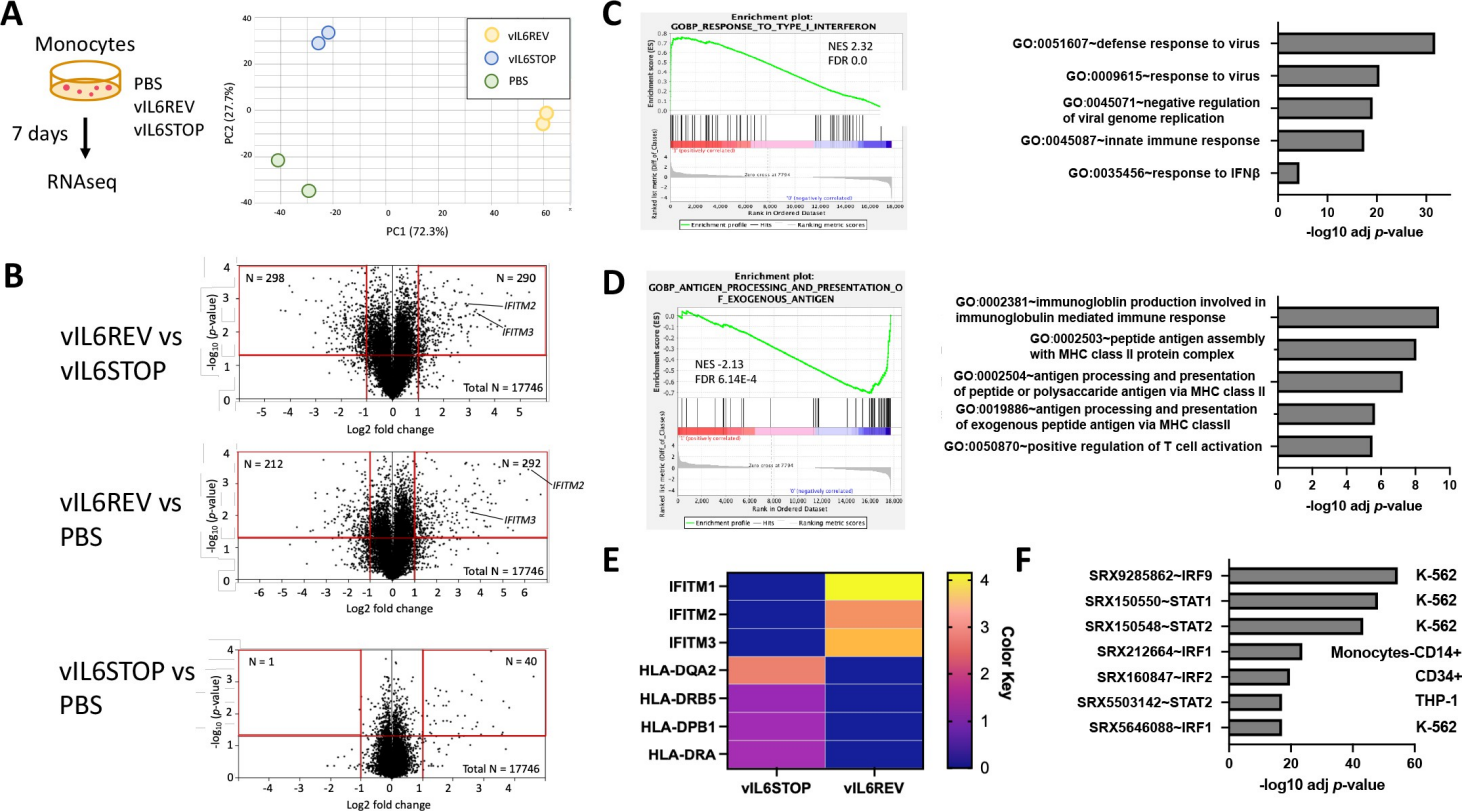
Differential gene expression analysis between wild-type KSHV and vIL-6STOP KSHV infected monocytes. (A) A schematic illustration of the KSHV infection experiment (Left). Monocytes were infected with vIL-6REV, vIL-6STOP, or mock-infected (PBS). At 7 dpi, cells were harvested for RNA-seq analysis (n = 2/group). Principal component analysis (PCA) of RNA-seq data from independent biological replicates is shown (Right). The number in parenthesis indicates the percentage of total variance explained by each PC. Colored dots represent individual samples infected with mock (PBS-treated), vIL-6REV, and vIL-6STOP. (B) Volcano plots for differentially expressed genes (log2 FC threshold = 1, *p*-value threshold = 0.05) between vIL-6REV and vIL-6STOP (Top), vIL-6 REV and mock (Middle) and vIL-6STOP and mock (Bottom). The log2 FC indicates the mean expression for each. The functional enrichment analysis of upregulated genes (C) and downregulated genes (D) in vIL-6REV- compared to vIL-6STOP-infected monocytes. DAVID functional enrichment analysis was performed, and the analysis results of the top 5 enriched pathways are shown (Right). The representative results of Gene Set Enrichment Analysis (GSEA) are shown (Left). (E) Heatmap of representative genes from enriched gene sets for upregulated IFN-inducible genes (C) or downregulated MHC-II genes (D) in vIL-6REV- compared to vIL-6STOP-infected monocytes. (F) Putative transcription factors that are responsible for differentially expressed genes between vIL-6 REV and vIL-6 STOP KSHV infection. The bar charts indicate *p* values for enrichment. The following parameters were used; Organism: Homo sapiens (hg38), Experiment type: ChiP (TFs and others), Cell type Class: Blood, and Threshold for significance: 50.

**Fig 5:**
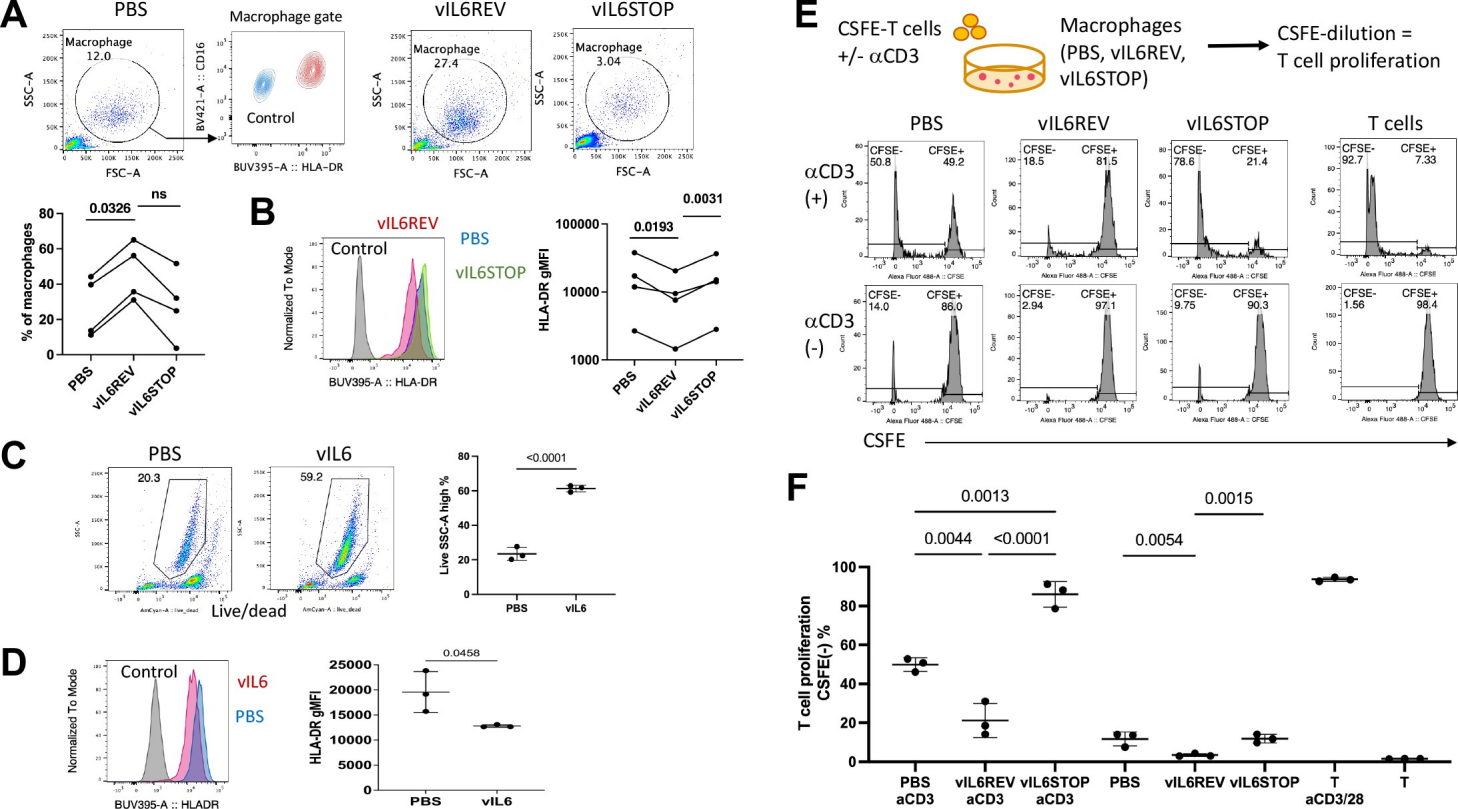
KSHV infection promotes macrophage differentiation with an immunosuppressive phenotype in a manner dependent on vIL-6 expression. Monocytes were infected with vIL-6REV or vIL-6STOP (MOI = 1), uninfected (PBS) and cultured for 7–14 days, or treated with vIL-6 (200 ng/ml in serum-free medium) for 9 days. Each sample was analyzed by flow cytometry with isotype control staining as a control. (A) The average frequency of macrophages in each culture (n = 3) is based on representative FSC-H and SSC-H profiles for uninfected (PBS) and vIL-6REV- and vIL-6STOP-infected samples. The macrophage gate was further analyzed for HLA-DR and CD16 expression to confirm typical macrophage phenotype. A representative overlay for the treated sample with unstained control (blue) and stained with antibodies for HLA-DR and CD16 (red). Data combine four independent experiments with different donor samples. Dots represent the average % of biological replicates (n = 2–3). (B) The average geometric mean fluorescent intensity (MFI) of HLA-DR for each macrophage sample with representative overlays is shown. Data combine four independent experiments with different donor samples. Dots represent the average HLA-DR gMFI of biological replicates (n = 2–3). (C) Representative FACS plots of live monocytes one week after vIL-6 treatment and the average frequencies (n = 3) are shown. (D) The average gMFI of HLA-DR for each macrophage sample in (C) with representative overlays are shown. Data represent two similar experiments. (E) An experimental design of T cell proliferation assay with macrophages (Top) and representative histograms of T cells (gated based on FSC-A and SSC-A) for CSFE are shown. T cells without or with anti-CD3/CD28 tetramer stimulation are used as controls. (F) The average frequencies of the CFSE-negative population determined as in (E) for each sample are shown (n = 3). *p* values shown are by ANOVA with a paired comparison with Tukey’s multiple comparison test. *p* < 0.05: statistically significant. Data represent two similar experiments.

To reveal the biological effect of vIL-6 expression in infected monocytes, we next conducted a transcriptomic analysis of *de novo* infected monocytes. To this end, total RNA was isolated from monocytes harvested 7 days post KSHV infection, and RNA-seq was performed (Fig 4A in the Left panel). By 7 dpi, the recovery of vIL-6REV-infected monocytes was higher than vIL-6STOP-infected monocytes, as shown later in Fig 5A, and they are consistent with increased Ki67^+^ expression (Fig 3B and 3C). Principal component analysis (PCA) demonstrated markedly distinct transcriptional profiles between vIL-6REV-infected and vIL-6STOP-infected monocytes (Fig 4A). Volcano plots depict the higher numbers of differentially regulated genes in vIL-6REV-infected monocytes (Fig 4B, e.g., relative to PBS control). To our surprise, vIL-6STOP infection had little impact on the transcriptional landscape of monocytes at 7 dpi, suggesting that sustaining STAT signaling activation may be important for cell reprogramming or vIL-6 is important for KSHV reactivation or maintaining persistent KSHV lytic replication. The phenotype is partly seen in reactivated iSLK cells ([30] and S5D Fig). Those two functions of vIL-6 are likely to be mutually exclusive.

Among the genes differentially expressed between vIL-6REV and vIL-6STOP infection, interferon-induced genes, such as *IFITM2* and *IFITM3*, are substantially upregulated in response to vIL-6REV infection (Fig 4B, Top panel). Consistently, Gene Set Enrichment Analysis (GSEA) also revealed that genes associated with host anti-viral innate responses were enriched in vIL-6REV infection (Figs 4C and S7A), while the pathways involving in antigen processing and presentation were significantly downregulated in vIL-6REV but not in vIL-6STOP infected monocytes (Figs 4D and S7B). While we expected to see upregulation of IFN-related genes in KSHV infected cells, down-regulation of MHC class II genes were unexpected. Representative upregulated IFN-inducible genes and down-regulation of MHC-II genes were visualized in the heatmap (Fig 4E). To identify the putative transcription factors that are responsible for vIL-6-driven gene regulation, we utilized bioinformatics tools to examine transcription factors whose binding are enriched on promoter regions of differentially transcribed genes. As shown in Fig 4F, interferon regulatory factors STAT1 and STAT2 were found to be putative transcription factors responsible for driving vIL-6-associated phenotypes. In support of this, we found that a subsets of STAT target genes were expressed slightly higher in vIL-6REV-infected monocytes compared to vIL-6STOP-infected monocytes (S7C Fig).

### KSHV infection generates macrophages with an immunosuppressive phenotype in a manner dependent on vIL-6 expression

Following the transcriptomic study that demonstrated transcriptional landscape changes in KSHV-infected macrophages, we next examined biological effects on monocytes after KSHV *de novo* infection on 7–14 dpi. For this, we compared the T-cell activation function between vIL-6REV and vIL-6STOP infected monocytes.

We showed in Fig 1E that the M2-like or suppressive macrophage differentiation program were triggered by 4 dpi. Consistent with this, monocytes in cultures showed features indicative of macrophages, such as being predominantly of large cellularity with higher FSC and SSC with HLA-DR and CD16 expression (Fig 5A). Consistent with the transcriptomic analysis in Fig 4D, the macrophages derived from vIL-6REV infection (vIL-6REV) expressed lower levels of HLA-DR compared to control groups (Fig 5B). To test the direct effect of vIL-6 in such phenotypic changes (absence of other viral genes), we generated functional recombinant vIL-6 protein, which is capable of activating STAT1 and 3 (S8 Fig). Monocytes were cultured in the presence of vIL-6 (200 ng/ml in serum-free medium) for 9 days, and the phenotype was analyzed by flow cytometry. As shown in Fig 5C, the overall monocyte viability was increased from 20% to 59% in the presence of vIL-6. In addition, monocytes recovered from the 9-day culture showed a trend of reduced HLA-DR expression (Fig 5D) compared to monocytes cultured without vIL-6 (PBS). The results suggested that vIL-6 treatment alone could induce phenotypic changes similar to vIL-6REV infection, perhaps due to continued STAT activations.

Finally, to evaluate T cell co-stimulatory capacity, CSFE-labeled allogeneic T cells were cultured with macrophages derived from monocytes mock-infected or infected with vIL-6REV or vIL-6STOP. T cell proliferation was then evaluated as CSFE-dilution by flow cytometry (Fig 5E, Top panel) in the presence or absence of anti-CD3 stimulation and with the T cell culture with anti-CD3/CD28 tetramer stimulation as a positive control. As shown in Fig 5E and summarized in Fig 5F, vIL-6REV infection significantly impaired T-cell activation function of infected macrophage, which is demonstrated by the decreasing CFSE-negative proliferated T cells in co-culture to (18.5%) compared with uninfected (50.8%) or vIL-6STOP-infection groups (78.6%) in the presence of anti-CD3 stimulation. Similar results were found in the cultures without anti-CD3 stimulation, with the exception that the extent of T cell proliferation was smaller. These observations are consistent with the transcriptional analysis in Fig 4, which collectively demonstrates that sustained vIL-6-driven host anti-viral response in infected monocytes induces an immune suppressive phenotype in differentiating macrophages.

### vIL-6 expression is critical for having KSHV replication in monocytes

**Fig 6:**
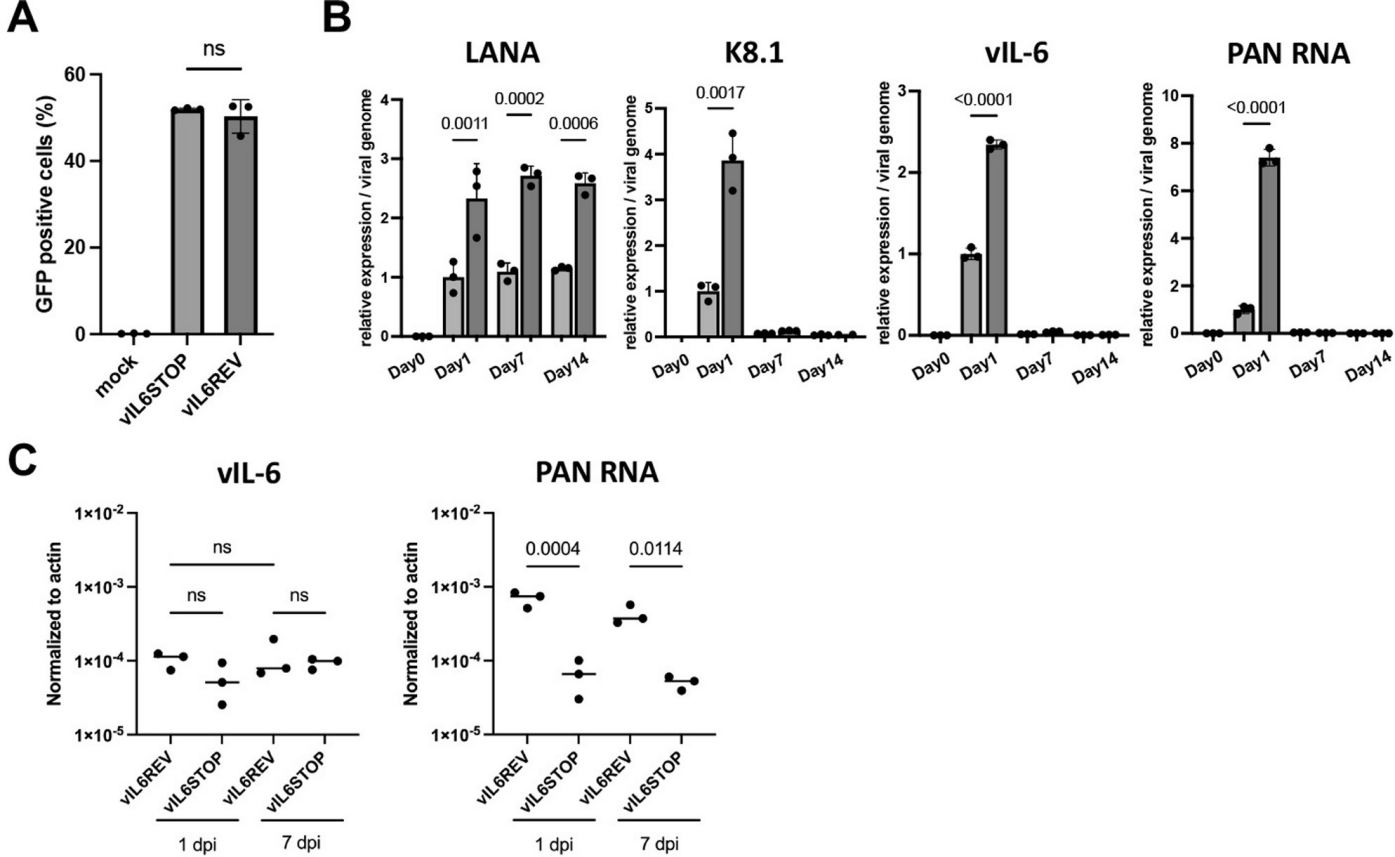
vIL-6 protein expression is important to sustain KSHV inducible gene expression. (A) iSLK cell lines were infected with vIL-6 REV or vIL-6 STOP rKSHV. The proportion of GFP-positive cells was determined by FACS analysis at 1 dpi. (B) Total RNA was isolated from vIL-6 REV or vIL-6 STOP-infected iSLK cells at 0, 1, 7, 14 dpi. LANA, K8.1, vIL-6, and PAN mRNA expression normalized with β-actin were determined in triplicates by qRT-PCR. Each mRNA expression was then normalized by the viral genome copy. *p* values shown are by unpaired Student’s *t*-test. (C) Monocytes were infected with vIL-6-REV or vIL-6 STOP rKSHV, and vIL-6 and PAN mRNA expression levels normalized to β-actin were measured in triplicates by qRT-PCR at 1 and 7 dpi. *p* values shown are by ANOVA with a paired comparison with Tukey’s multiple comparison test. *p* < 0.05: statistically significant. Data represent two similar experiments.

As shown in Fig 2, at 1 dpi, both vIL-6-REV and vIL-6-STOP KSHV induced indistinguishable host anti-viral responses predominant with STAT 1 and 3 activations. However, when constructing vIL-6STOP, we observed greatly reduced KSHV protein (K-Rta, K-bZIP, and LANA) expression upon reactivation (S5 Fig). Also, in Figs 4 and 5 we found that in the absence of vIL-6, KSHV infection in monocytes generated macrophages indistinguishable with those from uninfected monocytes. These discrepancies raise an important question of whether KSHV infection or replication was aborted in the absence of vIL-6 during de novo infection. To examine this, we infected iSLK cells with the same amount of vIL-6-REV and vIL-6-STOP KSHV (Fig 6A) and measured KSHV gene expression over time. To reveal the efficacies of KSHV gene transcription, we normalized transcription with KSHV genome copy in addition to cellular internal control. As shown in Fig 6B, the expression of LANA, one of the KSHV latent genes, was maintained for 2 weeks, suggesting that both vIL-6STOP and vIL-6REV KSHV infection was maintained in culture. However, we also noticed that vIL-6STOP frequently showed lower LANA, vIL-6, K8.1 mRNAs, and PAN RNA in total cell lysates during the establishment of vIL-6STOP infected iSLK cells. The results are consistent with previous report which demonstrated that vIL-6 positively regulate the expression of other viral lytic genes and contribute to virus production [30]. The notion is also supported by the gene expression analysis in *de novo* infected monocytes with vIL-6STOP and those with vIL-6REV. That is PAN RNA expression, which is required for late KSHV gene expression [33], was substantially lower in monocytes infected with vIL-6STOP even though vIL-6 RNA expression was comparable between vIL-6REV and vIL-6STOP infection at 1 and 7 dpi in monocytes (Fig 6C). Impaired reactivation potency in vIL-6STOP infected cells suggest that vIL-6 may play an important role in establishing the active latent chromatins for KSHV reactivation and furthermore in epigenetic reprogramming of transcriptional pathways in host monocytes. In fact, the mechanism is attributed to establishing trained immunity in innate immune cells [34], which may explain the dysregulation of functions in KSHV-infected monocytes.

In summary, this study demonstrates the preferential KSHV infection of monocytes and highlights the critical role of vIL-6 in expanding infected monocytes that facilitate a long-term virus reservoir. Monocytes play a key role in early infections by producing inflammatory cytokines and differentiating into subtypes that play distinct roles in the induction or resolution of inflammation [35,36]. Upon activation, some monocytes upregulate CD16 (FcgRIII), giving rise to CD14^+^CD16^+^ intermediate monocytes and CD14^-^CD16^+^ non-classical monocytes with a prolonged circulating lifespan. These CD16^+^ monocytes normally exhibit high HLA-DR expression for the capability of antigen presentation, transendothelial migration, and FcR-mediated phagocytosis for anti-viral response [37]. CD16^+^ monocytes also express the highest levels of TNFR2 (CD120b) [38], which can upregulate the anti-inflammatory cytokine IL-10 [39] and support either cell survival or death [40]. In this context, we observed an increase in CD14^+^CD16^+^ cells during early *de novo* KSHV infection (Figs 2E and 3C). We speculate that KSHV infects CD14^+^CD16^+^ monocytes by exploiting their FcR- and complement-mediated phagocytosis mechanism to expand reservoirs.

The vIL-6-driven sustained STAT1/3 activation may provide infected monocytes with a proliferative advantage over uninfected cells to increase their population size. Furthermore, vIL-6-driven lytic infection constitutively triggers host anti-viral responses such as IFN-pathway activation, which, in turn, may induce negative feedback to dampen inflammatory responses [41]. In the case of *Mycobacterium*-infected macrophages, Type-I IFN signaling decreases energy metabolism (i.e., glycolysis and mitochondrial functions) [42] and causes cell death [43]. In *Listeria-*infected macrophages, stress-induced p38 mitogen-activated protein kinases (MAPK) signaling enhances IFN-stimulated genes (ISGs). In macrophages, IL-10 production as a part of the IFN-induced regulatory mechanism has been shown to dampen the capability of T-cell activation and antigen presentation [44–46]. We show that the induction of a suppressive M2-like macrophage gene was induced as early as 4 dpi in our scRNA analysis (Fig 1). Consistent with this, earlier studies by other researchers also showed that KSHV infection blocks the differentiation of dendritic cells [47] and that STAT3 activation from KSHV infection induces a suppressive phenotype in dendritic cells and the THP-1 cell line [48–51]. Accumulation of such immunosuppressive macrophages may increase the risk of secondary infection or cause chronic infection, leading to frequent inflammation in the host; this mechanism may increase the risk of KSHV-associated disease development.

Similar hijacking strategies to exploit monocyte development and functions have also been reported in cytomegalovirus [22,52–54]. The current study was unique in that vIL-6STOP and vIL-6REV rKSHV infection system was employed to delineate the role of vIL-6 from that of the other KSHV-encoded multifunctional factors that are also known to down-regulate host immune responses [55–59]. Continued stimulation with recombinant vIL-6 protein also induced immune suppressive phenotype in primary monocytes, presumably through reprogramming of host transcriptions. However, further study is needed on whether vIL-6 is directly involved as a driver in the expression of ISGs under the host defense program in infected monocytes. Nonetheless, vIL-6 was critical for the maintaining KSHV episomes that can express inducible genes in infected monocytes. In the absence of vIL-6, KSHV replication was significantly impaired, and infected monocytes generated macrophages indistinguishable from those from uninfected monocytes at 7dpi. Understanding the mechanism in which vIL-6 hijacks the IFN-induced regulatory mechanism and utilizes it to promote cell proliferation would help develop therapies for KSHV-associated diseases.

With our single cell RNA-sequence study, we could not detect typical KSHV latent gene expression in infected monocytes. This is likely due to less abundance of transcripts compared to robustly induced lytic gene transcripts, and the limitation of sequence depth with the single-cell sequencing approach. In future studies, it is essential to determine whether KSHV establishes persistent infection or typical latent infection in macrophages or both to spread KSHV in neighboring/interacting immune cells for a life-long viral reservoir. Of note, KSHV is found in circulating monocytes and macrophages [10,12], and blood-derived KS-like spindle cells were also found to express macrophage markers [60]. Furthermore, a recent study reported an intriguing finding that KSHV-infected monocytes recruit, activate, and promote plasma cell differentiation of B cells via chemokines, leading to long-term latency in B cells. The study also provides compelling evidence that the monocyte abundance from malaria infection may underlie KSHV’s geographic disparity [61]. These findings shed light on a strategic sequential cell-specific infection program KSHV has adopted to exploit our immune system and establish life-long latent infection. Future studies are needed to investigate the roles of monocytes/macrophages as a crucial viral reservoir in patients with KSHV-associated diseases.

## Materials and methods

### Ethics statement

Peripheral blood mononuclear cells (PBMCs) were recovered from Leukoreduction Chambers (LRCs) from Apheresis Collections purchased from Vitalant Research Institute. Those Leukoreduction Chambers were to be discarded during the normal course of volunteer blood donations collected from healthy donor subjects using FDA-approved collection methods under an IRB-approved protocol at Vitalant Research Institute, San Francisco, CA, with written informed consent provided. Since these cells were not obtained for the purpose of experimentation and the donors are anonymous, use of these cells was not considered human subject research requiring Institutional Review Board approval.

### Cells

LRCs from de-identifiable healthy donors were purchased from Vitalant and exempt for IRB. PBMCs were prepared by a standard Ficoll gradient method. PBMCs with a 10–30% range of CD14^+^ monocyte content measured by flow cytometry were stored in liquid N2 and used for experiments. PBMCs were thawed in warm media, washed twice, counted with the Countess automated cell counter (ThermoFisher), and resuspended at 2-5x10^6^ viable cells/ml. Monocytes were isolated with EasySep^TM^ Human monocyte isolation kit (STEMCELL) according to the manufacturer’s protocol. PBMCs and CD14^+^ monocytes were cultured in RPMI1640 medium containing 10% FBS plus antibiotics (2–5 x 10^6^ cells/ml, 200 μL/well in a 96 well plate, 400 μL/well in a 48 well plate, or 1ml/well in a 24 well plate) supplemented with or without stimuli for various time as described in each figure legend.

### Virus preparation

Doxycycline-inducible rKSHV.219 producer iSLK.219 cell lines were maintained in DMEM supplemented with 10% FBS, 1% penicillin-streptomycin-L-glutamine solution, 10 μg/mL puromycin, 400 μg/mL hygromycin B, and 250 μg/mL G418. For virus preparation, iSLK.219 cell lines were stimulated with 0.3 mM Sodium Butylate and 1 μg/ml Doxycycline for 5 days. Recombinant KSHV particles were then purified after two serial ultracentrifugation steps from the culture supernatant of activated iSLK.219 cell lines. Briefly, the culture supernatant was centrifuged at 300 × g for 10 min and then passed through a 0.8-μm filter to remove cellular debris, and then viral particles were concentrated by ultracentrifugation at 25,000 rpm for 2 hrs at 4°C with a Beckman SW28 rotor. The viral precipitates were resuspended in 500 μL of DMEM along with the residual ∼500 μL media in the centrifuge tube and stored at −80°C until use.

### Quantification of viral copy number

Two hundred microliters of cell culture supernatant were treated with DNase I (12 μg/mL) for 15 min at room temperature to degrade unencapsidated DNA. This reaction was stopped by the addition of EDTA to 5 mM, followed by heating at 70°C for 15 min. Viral genomic DNA was purified using the QIAamp DNA Mini Kit according to the manufacturer’s protocol and eluted in 100 μL of buffer AE. Four microliters of the eluate were used for real-time qPCR to determine viral copy number, as described previously [62].

### Recombinant KSHV

To test the role of vIL-6, BAC16 lacking vIL-6 protein expression (vIL-6STOP) was generated by adding stop codons and mutating start codon in the vIL-6 reading frame with BAC16 (vIL-6STOP) (S5 Fig). Long primer pairs were used to prepare transfer DNA fragment for recombinant by amplifying Kanamycin cassette from the pEP-Kan plasmid. The amplified DNA fragment was transformed into BAC16 KSHV containing *E*.*coli* for recombination. Mutation and surrounding junction sequences were confirmed by amplifying the genomic region, and the amplified DNA fragment was gel-purified and directly sequenced. We also generated a revertant BAC16 with vIL-6 protein expression (vIL-6REV) by changing the sequence of vIL-6STOP back to the original wild-type sequence with BAC-recombination.

### KSHV *de novo* infection

KSHV infection was conducted at MOI = 1. Briefly, PBMCs or monocytes (1–2 x 10^6^ cells) were suspended in RPMI 1640 medium containing 10% intact FBS (not heat inactivated) supplemented with 8 μg/ml polybrene and an appropriate amount of rKSHV.219, vIL-6REV, or vIL-6STOP rKSHV in PBS (less than 10% of total volume) or PBS alone (i.e., as mock infection) was added to the culture. Cells were kept in the same culture medium until analysis.

### Single-cell RNA-seq

2 x 10^6^ PBMCs were infected with rKSHV.219 at MOI = 1, washed and fixed with paraformaldehyde according to the 10x genomics cell preparation guide (CG000053) at various time points (day 0, 1, 2, and 4) after infection. Single-cell suspensions (10^4^ cells) were submitted to the UC Davis Comprehensive Cancer Center (UCDCCC) Genomics Shared Resource for processing on the Chromium Controller (10x Genomics) for cell partitioning into individual GEMs (gel bead-in-emulsion), generation of barcoded cDNAs with unique molecular identifiers (UMIs), and construction of barcoded sequencing libraries were constructed using the Chromium Single Cell 3’ Reagent Kits v2 (10x Genomics). The libraries were then multiplexed and sequenced (∼250 million mapped reads per sample) on an Illumina NovaSeq 6000 sequencing system. Single-cell data were analyzed with the Cell Ranger v2.1 pipeline (10x Genomics). The pipeline included alignment to the hg38 human reference genome and human herpesvirus 8 strain (GQ994935.1) reference genome with STAR [63], Uniform Manifold Approximation and Projection (UMAP), and K-means clustering. Read count matrices obtained from the pipeline were normalized using the log normalization method from the “Seurat” R package [64]. We investigated the association between genes using the Pearson product-moment correlation coefficient. To compute the correlation coefficient and p-value, we utilized the cor.test() function from the stats package in R.

### mRNA-seq

RNA was prepared from infected monocytes using a QIAGEN RNeasy Miniprep kit according to the manufacturer’s protocol and submitted to the UCDCCC GSR for mRNA-seq analysis. Indexed, stranded mRNA-seq libraries were prepared from total RNA (100 ng) using the KAPA Stranded mRNA-Seq kit (Roche) according to the manufacturer’s standard protocol. Libraries were pooled and multiplex sequenced on an Illumina NovaSeq 6000 System (150-bp, paired-end, >25 × 10^6^ reads per sample). The RNA-Seq data was analyzed using a STAR-HTSeq-DESeq2 pipeline. Raw sequence reads (FASTQ format) were mapped with STAR to the reference human genome assembly (GRCh38/hg38, GENCODE release 36) and quantified with HTSeq [65]. The resulting data were first filtered by average normalized count data >1, and volcano plots production and listing differentially expressed genes was performed by Subio Platform ver. 1.24 (Subio Inc., Amami, Japan). We employed p-values using Student’s t-test to visualize the differentially expressing genes. For transcription factor analysis, differentially expressed genes were submitted to Chip-Atlas to analyze common regulators and to predict transcription factor binding [66].

### RT-qPCR

Cells were washed with PBS, and total RNA was extracted using the Quick-RNA miniprep kit (Zymo Research, Irvine, CA, USA). A total of 1 μg of RNA was incubated with DNase I for 15 and reverse transcribed with the High-Capacity cDNA Reverse Transcription Kit (Thermo Fisher, Waltham, MA USA). The resulting cDNA was used for qPCR. SYBR Green Universal master mix (Bio-Rad) was used for qPCR according to the manufacturer’s instructions. Each sample was normalized to β-actin RNA, and the duct fold change method was used to calculate relative quantification. All reactions were run in triplicate using primers specific for KSHV ORFs designed by Fakhari and Dittmer [67].

### Western blotting

Cells were washed with PBS, lysed in lysis buffer (50 mM Tris-HCl [pH 6.8], 2% SDS, 10% glycerol) and boiled for 3min. The protein concentrations of the lysates were quantified with a BCA Protein Assay Kit (Thermo Fisher). Protein samples were separated by SDS-PAGE using 10% agarose gel and transferred to transfer membranes (Millipore-Sigma, St. Louis, MO, USA), which were incubated in 5% nonfat milk (A600669, Sangon) at room temperature for 2 hours. The membrane was incubated with the primary antibody at 4°C overnight or at room temperature for 2 hours. The membrane was then incubated with horseradish-peroxidase-conjugated secondary antibody or Alexa-647-conjugated secondary antibody at 25°C for 1 hour as described previously [68]. Anti-LANA antibody was purchased from Millipore-Sigma (St. Louis, MO, USA) and an Anti-K-Rta antibody was described previously [69].

### Phosphoflow PBMC CyTOF

This assay was performed by the Human Immune Monitoring Center at Stanford University. 2 x 10^6^ PBMCs were infected with rKSHV.219, washed twice with PBS, and then fixed with paraformaldehyde. Cells were washed twice with CyFACS buffer (PBS supplemented with 2% BSA, 2 mM EDTA, and 0.1% sodium azide) and stained for 30 min at room temperature with 20 mL of surface antibody cocktail. Cells were washed twice with CyFACS, permeabilized with 100% methanol, and kept at -80C overnight. The next day, cells were washed with CyFACS buffer and resuspended in a 20 mL intracellular antibody cocktail in CyFACS for 30 min at room temperature before washing twice in CyFACS. Cells were resuspended in a 100 mL iridium-containing DNA intercalator (1:2000 dilution in 2% PFA in PBS) and incubated at room temperature for 20 min. Cells were washed once with CyFACS buffer and twice with MilliQ water. Cells were diluted to 750x10^5^ cells/mL in MilliQ water and acquired on CyTOF. Data analysis was performed using FlowJo v10.8.1 by gating on intact cells based on the iridium isotopes from the intercalator, then on singlets by Ir191 vs cell length followed by cell subset-specific gating as described in S2 Fig.

### Flow cytometry

mAbs and isotype-matched controls were purchased from BD Bioscience (BUV395 conjugated HLA-DR, BUV395 conjugated to CD11c, PerCP-Cy5.5 conjugated to CD123, PE conjugated to CD11c, Alexa Fluor 647 conjugated to pSTAT-1, and Alexa Fluor 647 conjugated to pSTAT-3), and Biolegend (APC conjugated CD163, BV421 conjugated to CD16, PE-Cy7 conjugated to Ki67, APC conjugated to PD-L1, APC conjugated to CD14, and Alexa Fluor 488 conjugated to CD16). Cells in culture were washed twice with PBS and resuspended in FACS buffer (PBS supplemented with 1% FBS) at 1x10^7^ cells/ml. 50 uL cells per well were stained with Aqua LIVE/DEAD cell viability dye (Invitrogen, Carlsbad, CA) according to the manufacturer’s instructions, washed with FACS Buffer, and then stained for 45 min at room temperature with antibodies. For intracellular staining of pSTAT1 and pSTAT3, cells were fixed with paraformaldehyde, permeabilized with methanol, and kept at -80 C overnight. The cells were washed with FACS buffer and stained with pSTAT-1 Alexa Fluor 647 and pSTAT-3 Alexa Fluor 647. Cells were washed three times with FACS buffer and resuspended in 200 uL FACS buffer. For typical experiments, 10^5^ PBMCs per sample were collected using DIVA 6.0 software on a Fortessa flow cytometer (BD Biosciences) at the UCDCCC Flow Cytometry Shared Resource. Data analysis was performed using FlowJo v10.8.1 by gating on live cells based on forward versus side scatter profiles, then on singlets using forward scatter area versus height, followed by dead cell exclusion using Aqua LIVE/DEAD viability dye, and then cell subset-specific gating.

### T cell proliferation assay

Monocytes were infected with vIL-6REV or vIL-6STOP or mock-infected (PBS). Macrophages derived from KSHV-infected monocyte cultures were recovered at 7 dpi after being washed with PBS twice, followed by gentle pipetting and suspended in RPMI1640 medium containing 10% FBS. T cells were isolated from PBMCs using the EasySep^TM^ Human CD3 positive selection kit II (STEMCELL) according to the manufacturer’s protocol. T cells were stained in PBS with the CellTrace^TM^ CFSE Cell Proliferation Kit (ThermoFisher) according to the manufacturer’s protocol and suspended in RPMI1640 medium containing 10% FBS. CSFE-labeled CD3+ T cells (10^4^) were incubated with 10^3^ macrophages from KSHV-infected monocyte cultures in 200 μL/well in a 96-well U-bottom plate, without or with 100 ng/ml anti-CD3 antibody (BD Bioscience) for 5 days. For positive and negative controls, T cells were stimulated with ImmunoCult Human CD3/28 T cell activator (Stemcell) or left unstimulated. Cells were recovered from cultures and analyzed using a BD Accuri^TM^ flow cytometer. Data analysis was performed using FlowJo v10.8.1 by gating lymphocytes based on forward versus side scatter profiles. The average frequencies of the CFSE-negative population were determined based on the negative control.

### Statistical analysis

Statistical analyses were performed using GraphPad Prism 9.4.1 software. Results are shown as mean ± SD with dots representing individual measurements. Statistical significance was determined by Student’s t-test, ratio paired t-test, or one-way ANOVA with Tukey’s multiple comparison test, and correction for false discovery rate (FDR) as described in each figure legend. FDR corrected p < 0.05 was considered statistically significant.

## Supporting information

S1 Table List of differentially expressed genes in each cell cluster in the single cell RNA-sequencing. (XLSX)

S1 Fig Assignment of immune cell subsets in KSHV-infected PBMCs. (A) Immune cell clustering. PBMCs were infected with rKSHV.219 at MOI = 1, fixed at various time points (pre-infection, day 1, 2, and 4) after infection, and subjected to 10x Genomics Chromium scRNA-seq analysis. To identify immune cell subsets, UMAP visualization was applied to scRNA-seq data set using Loupe Browser 5 software. UMAP visualizes 9 PBMC clusters in 1 day post infection (dpi) sample based on differentially expressed gene listed in S1 Table. Clusters were categorized into major immune cell subsets based on lineage-specific gene expression of *KLRD1*, *CD14*, *APOBEC3A*, *VMO1*, *CD79A*, and *CD3E* genes for NK cells, monocytes, intermediate monocytes, non-classical monocytes, B cells, and T cells, respectively. **(B) Immune cell composition.** Composition (%) of each immune cell subset based on lineage-specific gene expression of *KLRD1*, *CD14*, *CD79A*, and *CD3E* genes for NK cells, monocytes including intermediate and non-classical monocytes, B cells, and T cells, respectively, is shown for pre-infection, and 1-, 2-, and 4-dpi samples. Total single cell counts are shown.
(TIF)

S2 Fig KSHV gene expression identified in single PBMCs. **(A) KSHV transcripts in each immune cell type.** PBMCs were infected with rKSHV.219 at MOI = 1, fixed at various time points (pre-infection, day 1, 2, and 4) after infection, and subjected to 10x Genomics Chromium scRNA-seq analysis. Number of single cells with detectable levels of each KSHV gene expression are shown for each immune cell subset (Monocytes, B cells, T cells, and NK cells) in pre-infection, and 1-, 2-, and 4-dpi PBMC samples. **(B) The frequencies of K2 and ORF16 transcripts.** The frequency (%) of single cells with detectable K2 and ORF16 gene expression in each immune cell subset (Monocytes, B cells, T cells and NK cells) of 1-, 2-, and 4-dpi PBMC samples are shown.
(TIF)

S3 Fig Tracking KSHV-infected cells by eGFP reporter expression. **(A) Representative eGFP reporter expression.** PBMCs were infected with rKSHV.219 at MOI = 1. At 1 day after infection, cells were stained with LIVE/DEAD Aqua, followed by staining with antibodies against CD14-BUV-395, HLA-DR-PE Texas Red, CD11c-PE-Cy7, CD3-BUV737, and CD19-BV421, and analyzed by BD Fortessa. KSHV infected cells were then monitored by eGFP reporter expression under the control of human EF-1 alpha promoter. Representative FACS plots for eGFP expression of live singlet PBMCs either left uninfected or KSHV-infected, followed by staining with antibodies (Ab) against indicated cell surface markers or unstained as control. FACS plot overlay of Ab-stained (red) and unstained control (blue) KSHV-infected samples are also shown in (B) and (C). **(B) Histograms.** The histogram in the right panel shows the percentage of eGFP(+) and eGFP(-) population among the gated CD14+ cells in the left panel (16.1% among live singlet PBMCs). 65.6% of CD14+ cells are positive for eGFP expression and infected with KSHV. **(C) Frequent KSHV infections in monocytes.** The majority of eGFP(+) cells express HLA-DR and CD11c, but not the lineage markers for B cells (CD19) or T cells (CD3).
(TIF)

S4 Fig The majority of CD14+ monocytes express high levels of vIL6 after KSHV infection. **(A) Representative FACS plots with vIL-6 staining.** PBMCs were infected with rKSHV.219 at MOI = 1. At 2 days after infection, cells were stained with LIVE/DEAD Fixable Far Red Dead Cell stain kit, followed by Fixation with 1% PFA/PBS for 10 min at room temperature and permeabilization with 0.2% Triton X-100 and 1% BSA. Cells were then stained with CD14-PE with or without rabbit anti-vIL-6 antibody, followed by anti-rabbit antibody conjugated with FITC, and analyzed by BD Canto. Representative FACS plots for vIL6 intracellular staining (Top panel) and control staining without anti-vIL6 antibody (Bottom panel) for CD14+ cells. % of CD14+ cell gates among live PBMCs are shown. **(B) Histograms.** Histograms of vIL6 intracellular staining for CD14(+) population (Left panel) and Non-CD14(+) population (Right panel). Each gated population was further sub-gated based on the expression levels of vIL6: vIL6 High, vIL6 Int (intermediate), and vIL6 Low. % of each sub-gated population is shown.
(TIF)

S5 Fig Preparation of vIL-6 STOP recombinant KSHV. **(A) Primer sequences.** Long primer pairs were used to prepare transfer DNA fragments for recombinant by amplifying the kanamycin cassette from pEP-Kan plasmid. The position of the intended mutation is underlined, primer sequence annealed to Kanamycin plasmid template is shown in bold italic. The amplified DNA fragment was transformed into BAC16 KSHV containing *E*.*coli* for recombination. The kanamycin cassette was removed by inducing I-SceI and red recombination. Mutation and surrounding junction sequences were confirmed by amplifying the genomic region, and the amplified DNA fragment was gel-purified and directly sequenced. **(B) DNA sequence.** DNA sequence of one of three vIL-6 STOP clones is shown. Two other clones are identical to vIL-6 STOP #2–7. **(C) Amino acid sequence alignment.** Intended mutation and stop codon is shown. To prepare the revertant clone, we used vIL-6 STOP#2–7 as a template to insert wild-type sequences by repeating the same procedure. **(D) Immunoblotting.** The iSLK cells infected with indicated recombinant KSHV were stimulated for reactivation with doxycycline (1 μg/mL) and TPA for 48 hours. Total cell lysates were used to probe for KSHV proteins. Indicated antibodies were used for immunoblotting, and cellular β-action was used as loading control. **(E) KSHV virion production in culture media.** Encapsidated KSHV genomic DNA copies were measured by qPCR. Viral DNA copy per microliter is shown.
(TIF)

S6 Fig CyTOF phospho panel and the gating strategy. A gating strategy to identify immune cell subsets is shown.
(TIF)

S7 Fig vIL-6 dependent transcriptional reprogramming of macrophages (A and B) Gene Set Enrichment Analysis (GSEA) for vIL6REV-infected vs mock-infected macrophage transcriptomic landscape. The functional enrichment analysis of upregulated genes (A) and downregulated genes (B) in vIL6REV- compared to mock-infected (PBS treated) monocytes are shown. DAVID functional enrichment analysis was performed, and the top 5 enriched pathway are shown (Right). The representative results of GSEA are shown (Left). **(C) Heatmap.** Heatmap of representative STAT3 downstream genes expression between vIL6STOP-infected monocytes and vIL6REV-infected monocytes. The Log2 fold change (FC) is shown when genes expression with vIL6STOP-infected monocytes is set to 1.
(TIF)

S8 Fig Validation of recombinant vIL6. **(A) Recombinant vIL-6 protein**. Recombinant vIL-6-Flag protein was produced in insect cell lines, and SDS-PAGE confirmed the purity. A gel image with molecular weight markers is shown. **(B) Ligand activity**. 293T and iSLK ORF57-wt cell lines were stimulated with 200 ng/ml recombinant vIL-6 along with controls, TNFα, IFNα, and human (h)IL-6, or mock-treated. STAT1 and 3 activations were studied by Western blotting with specific antibodies for total STAT1, STAT1 pY701, total STAT3, STAT3 pY705, and IkBα as controls. Ponceau staining for each well are shown as loading control. **(C) vIL-6 Cysteine mutant**. To validate vIL-6 activity, wild type (Wt) or mutant (C->A) was generated. Arrows indicate the location of amino acid residues where mutations are introduced. **(D) Coomassie staining**. Wt and C/A mutant vIL-6 proteins were expressed in 293FT cell lines, and the purity of recombinant proteins was examined by SDS-PAGE. **(E) Confirmation of signaling activation activity**. iSLK and THP-1 cell lines were stimulated with 200 ng/ml recombinant Wt or C/A mutant vIL6, or mock-treated. STAT3 activation was studied by Western blotting with specific antibodies for total STAT3, STAT3 pY705, and b-actin as controls. Wt but not C/A mutant triggered STAT3 activation.
(TIF)
