# Supplementary figures and images for: Virally encoded interleukin-6 facilitates KSHV replication in monocytes and induction of dysfunctional macrophages
Source: PLoS Pathog. 2023 Oct 26;19(10):e1011703. doi: 10.1371/journal.ppat.1011703 (PMC10602306; doi:10.1371/journal.ppat.1011703)

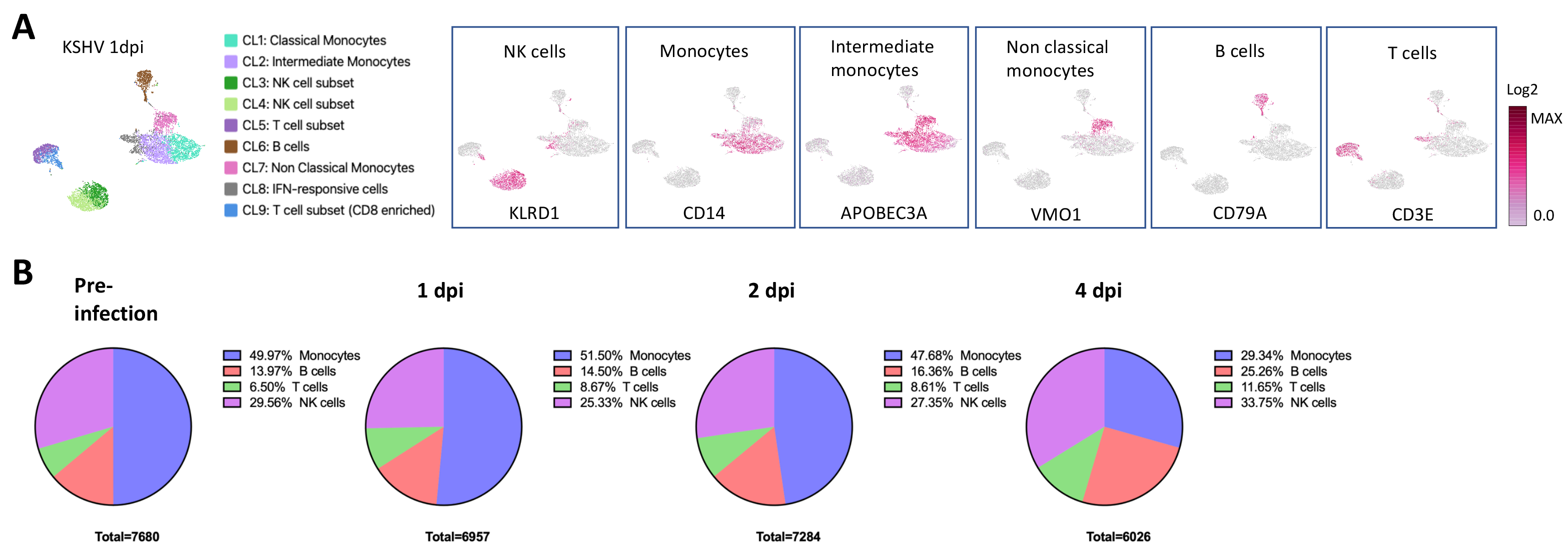

Supplement: S1 Fig — PBMCs were infected with rKSHV.219 at MOI = 1, fixed at various time points (pre-infection, day 1, 2, and 4) after infection, and subjected to 10x Genomics Chromium scRNA-seq analysis. To identify immune cell subsets, UMAP visualization was applied to scRNA-seq data set using Loupe Browser 5 software. UMAP visualizes 9 PBMC clusters in 1 day post infection (dpi) sample based on differentially expressed gene listed in S1 Table. Clusters were categorized into major immune cell subsets based on lineage-specific gene expression of KLRD1, CD14, APOBEC3A, VMO1, CD79A, and CD3E genes for NK cells, monocytes, intermediate monocytes, non-classical monocytes, B cells, and T cells, respectively. (B) Immune cell composition. Composition (%) of each immune cell subset based on lineage-specific gene expression of KLRD1, CD14, CD79A, and CD3E genes for NK cells, monocytes including intermediate and non-classical monocytes, B cells, and T cells, respectively, is shown for pre-infection, and 1-, 2-, and 4-dpi samples. Total single cell counts are shown. (TIF) [file ppat.1011703.s002.tif]

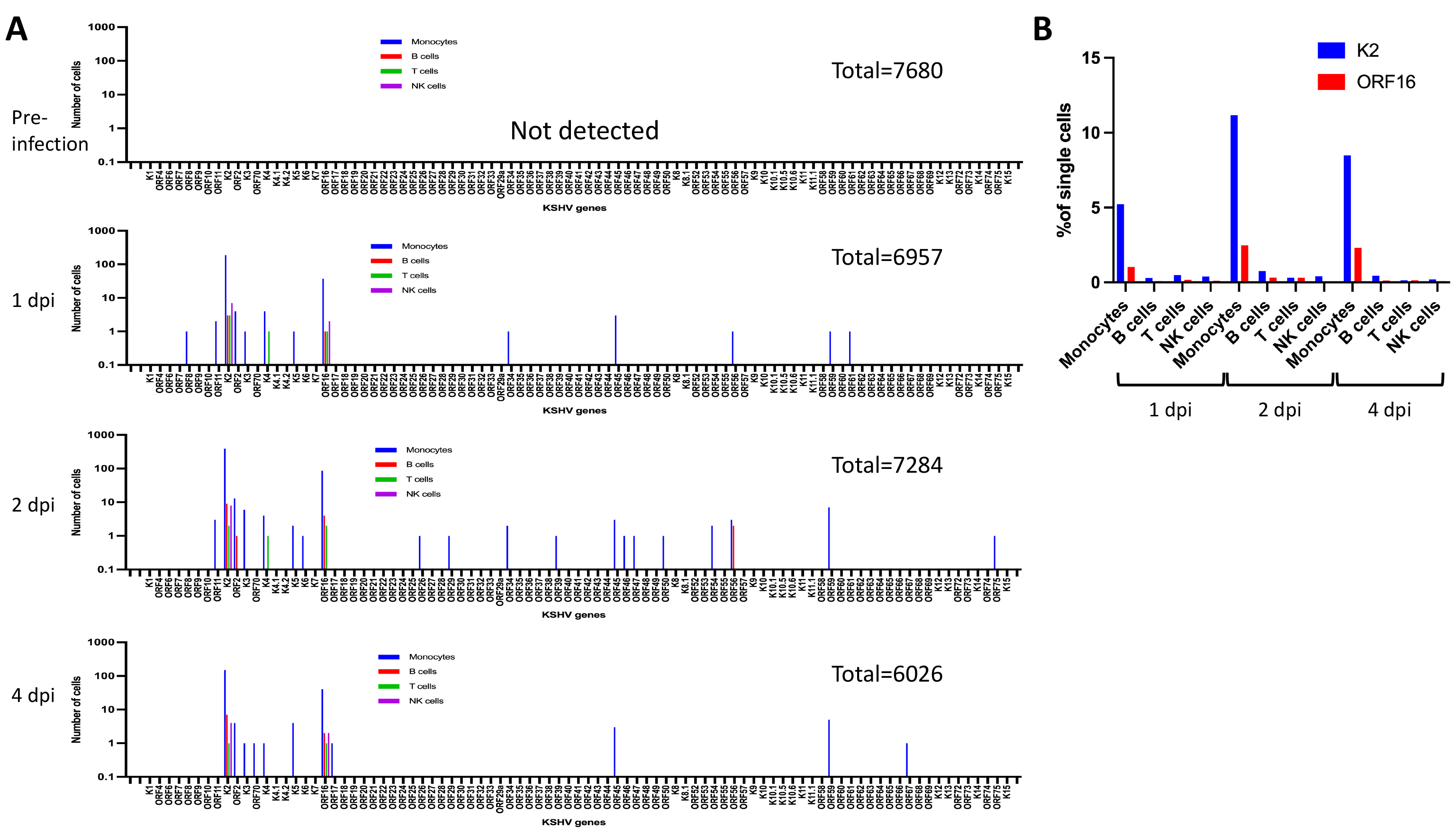

Supplement: S2 Fig — (A) KSHV transcripts in each immune cell type. PBMCs were infected with rKSHV.219 at MOI = 1, fixed at various time points (pre-infection, day 1, 2, and 4) after infection, and subjected to 10x Genomics Chromium scRNA-seq analysis. Number of single cells with detectable levels of each KSHV gene expression are shown for each immune cell subset (Monocytes, B cells, T cells, and NK cells) in pre-infection, and 1-, 2-, and 4-dpi PBMC samples. (B) The frequencies of K2 and ORF16 transcripts. The frequency (%) of single cells with detectable K2 and ORF16 gene expression in each immune cell subset (Monocytes, B cells, T cells and NK cells) of 1-, 2-, and 4-dpi PBMC samples are shown. (TIF) [file ppat.1011703.s003.tif]

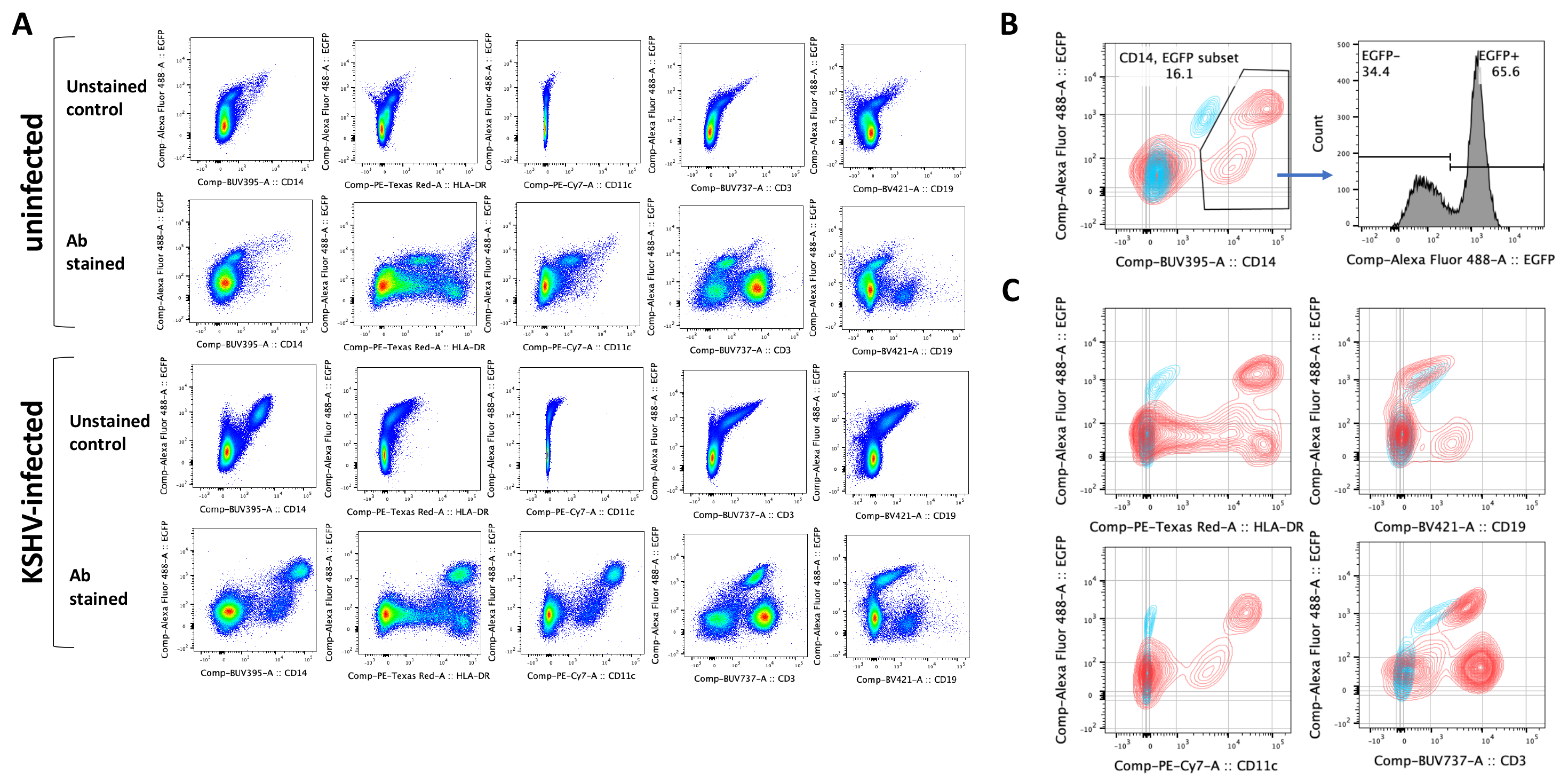

Supplement: S3 Fig — (A) Representative eGFP reporter expression. PBMCs were infected with rKSHV.219 at MOI = 1. At 1 day after infection, cells were stained with LIVE/DEAD Aqua, followed by staining with antibodies against CD14-BUV-395, HLA-DR-PE Texas Red, CD11c-PE-Cy7, CD3-BUV737, and CD19-BV421, and analyzed by BD Fortessa. KSHV infected cells were then monitored by eGFP reporter expression under the control of human EF-1 alpha promoter. Representative FACS plots for eGFP expression of live singlet PBMCs either left uninfected or KSHV-infected, followed by staining with antibodies (Ab) against indicated cell surface markers or unstained as control. FACS plot overlay of Ab-stained (red) and unstained control (blue) KSHV-infected samples are also shown in (B) and (C). (B) Histograms. The histogram in the right panel shows the percentage of eGFP(+) and eGFP(-) population among the gated CD14+ cells in the left panel (16.1% among live singlet PBMCs). 65.6% of CD14+ cells are positive for eGFP expression and infected with KSHV. (C) Frequent KSHV infections in monocytes. The majority of eGFP(+) cells express HLA-DR and CD11c, but not the lineage markers for B cells (CD19) or T cells (CD3). (TIF) [file ppat.1011703.s004.tif]

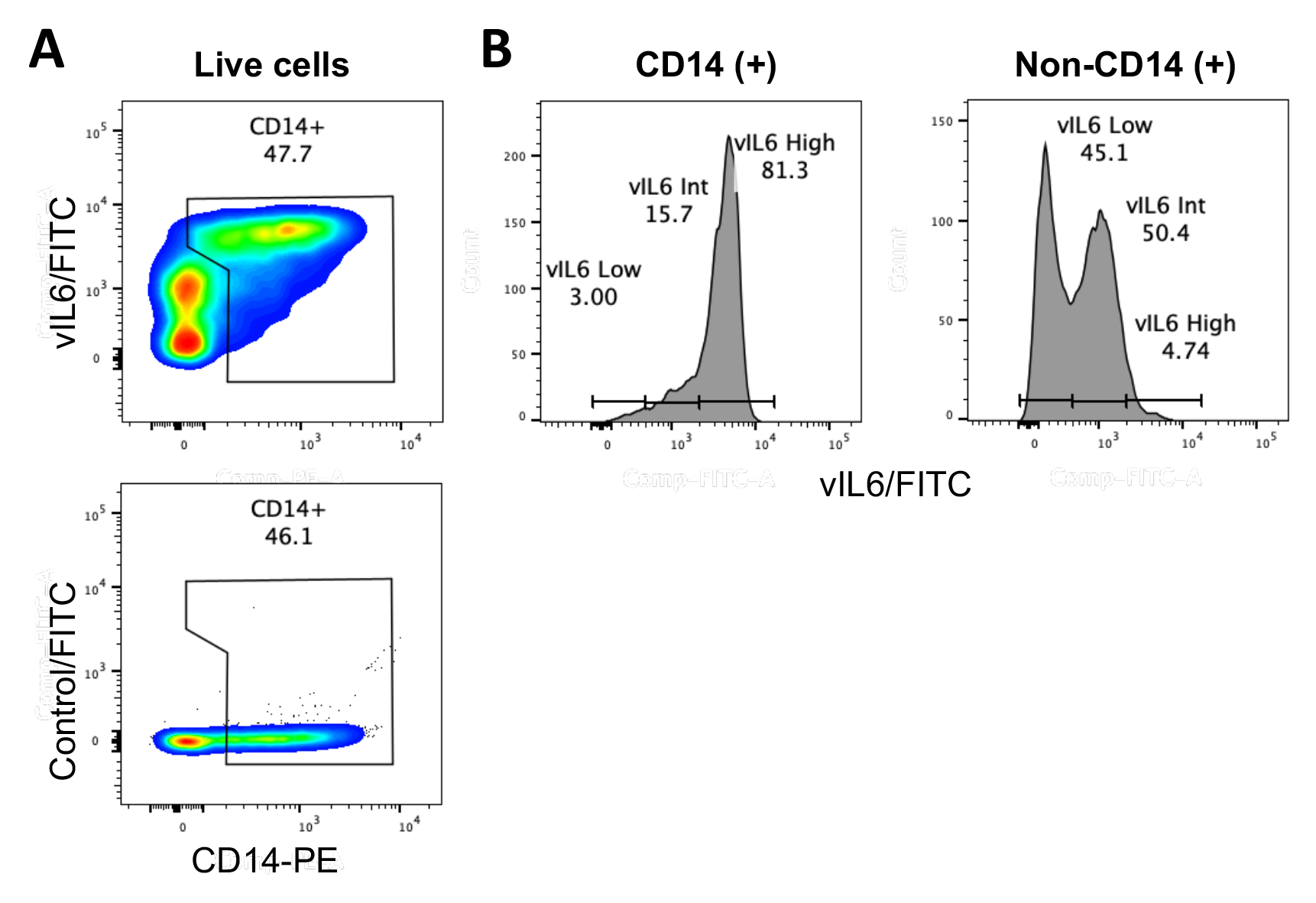

Supplement: S4 Fig — (A) Representative FACS plots with vIL-6 staining. PBMCs were infected with rKSHV.219 at MOI = 1. At 2 days after infection, cells were stained with LIVE/DEAD Fixable Far Red Dead Cell stain kit, followed by Fixation with 1% PFA/PBS for 10 min at room temperature and permeabilization with 0.2% Triton X-100 and 1% BSA. Cells were then stained with CD14-PE with or without rabbit anti-vIL-6 antibody, followed by anti-rabbit antibody conjugated with FITC, and analyzed by BD Canto. Representative FACS plots for vIL6 intracellular staining (Top panel) and control staining without anti-vIL6 antibody (Bottom panel) for CD14+ cells. % of CD14+ cell gates among live PBMCs are shown. (B) Histograms. Histograms of vIL6 intracellular staining for CD14(+) population (Left panel) and Non-CD14(+) population (Right panel). Each gated population was further sub-gated based on the expression levels of vIL6: vIL6 High, vIL6 Int (intermediate), and vIL6 Low. % of each sub-gated population is shown. (TIF) [file ppat.1011703.s005.tif]

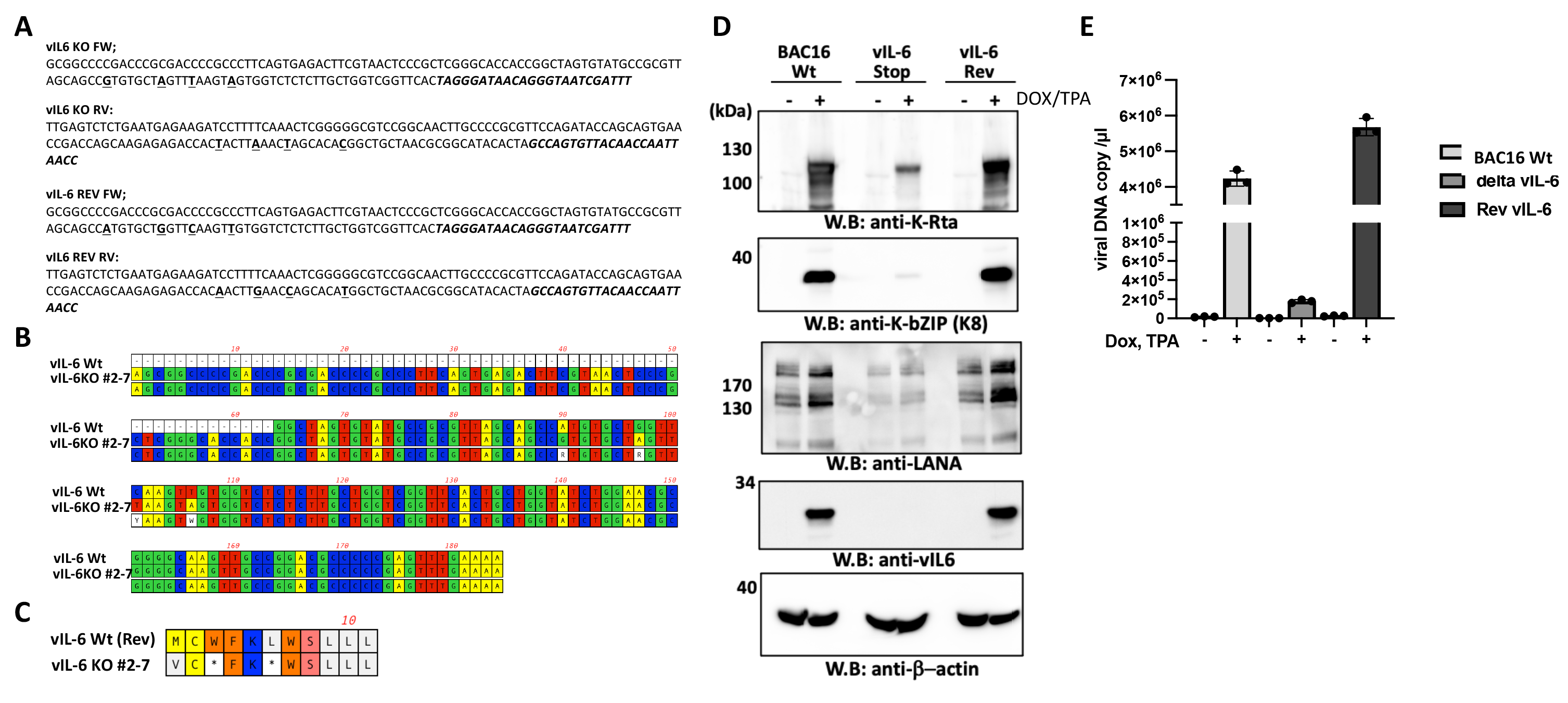

Supplement: S5 Fig — (A) Primer sequences. Long primer pairs were used to prepare transfer DNA fragments for recombinant by amplifying the kanamycin cassette from pEP-Kan plasmid. The position of the intended mutation is underlined, primer sequence annealed to Kanamycin plasmid template is shown in bold italic. The amplified DNA fragment was transformed into BAC16 KSHV containing E.coli for recombination. The kanamycin cassette was removed by inducing I-SceI and red recombination. Mutation and surrounding junction sequences were confirmed by amplifying the genomic region, and the amplified DNA fragment was gel-purified and directly sequenced. (B) DNA sequence. DNA sequence of one of three vIL-6 STOP clones is shown. Two other clones are identical to vIL-6 STOP #2–7. (C) Amino acid sequence alignment. Intended mutation and stop codon is shown. To prepare the revertant clone, we used vIL-6 STOP#2–7 as a template to insert wild-type sequences by repeating the same procedure. (D) Immunoblotting. The iSLK cells infected with indicated recombinant KSHV were stimulated for reactivation with doxycycline (1 μg/mL) and TPA for 48 hours. Total cell lysates were used to probe for KSHV proteins. Indicated antibodies were used for immunoblotting, and cellular β-action was used as loading control. (E) KSHV virion production in culture media. Encapsidated KSHV genomic DNA copies were measured by qPCR. Viral DNA copy per microliter is shown. (TIF) [file ppat.1011703.s006.tif]

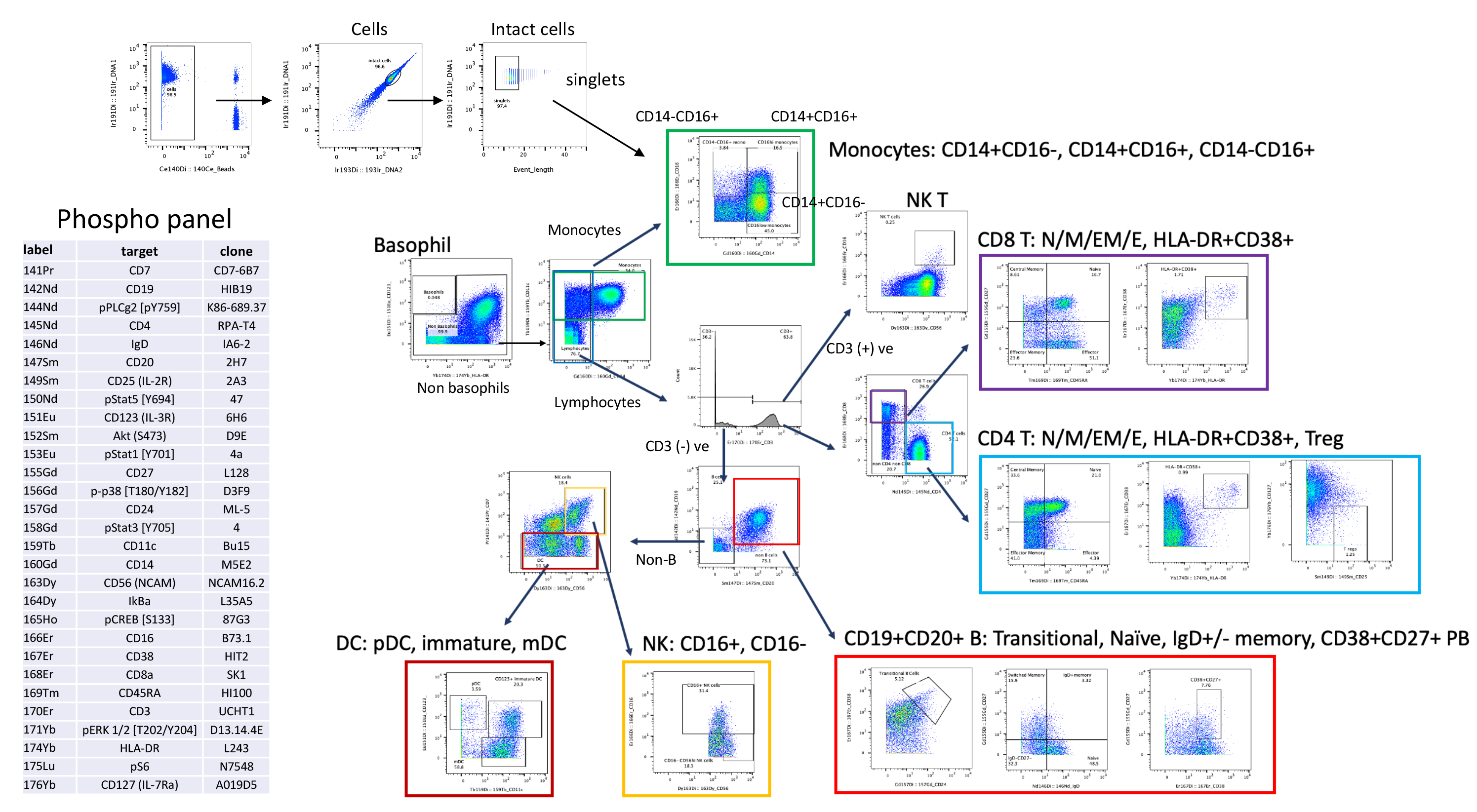

Supplement: S6 Fig — A gating strategy to identify immune cell subsets is shown. (TIF) [file ppat.1011703.s007.tif]

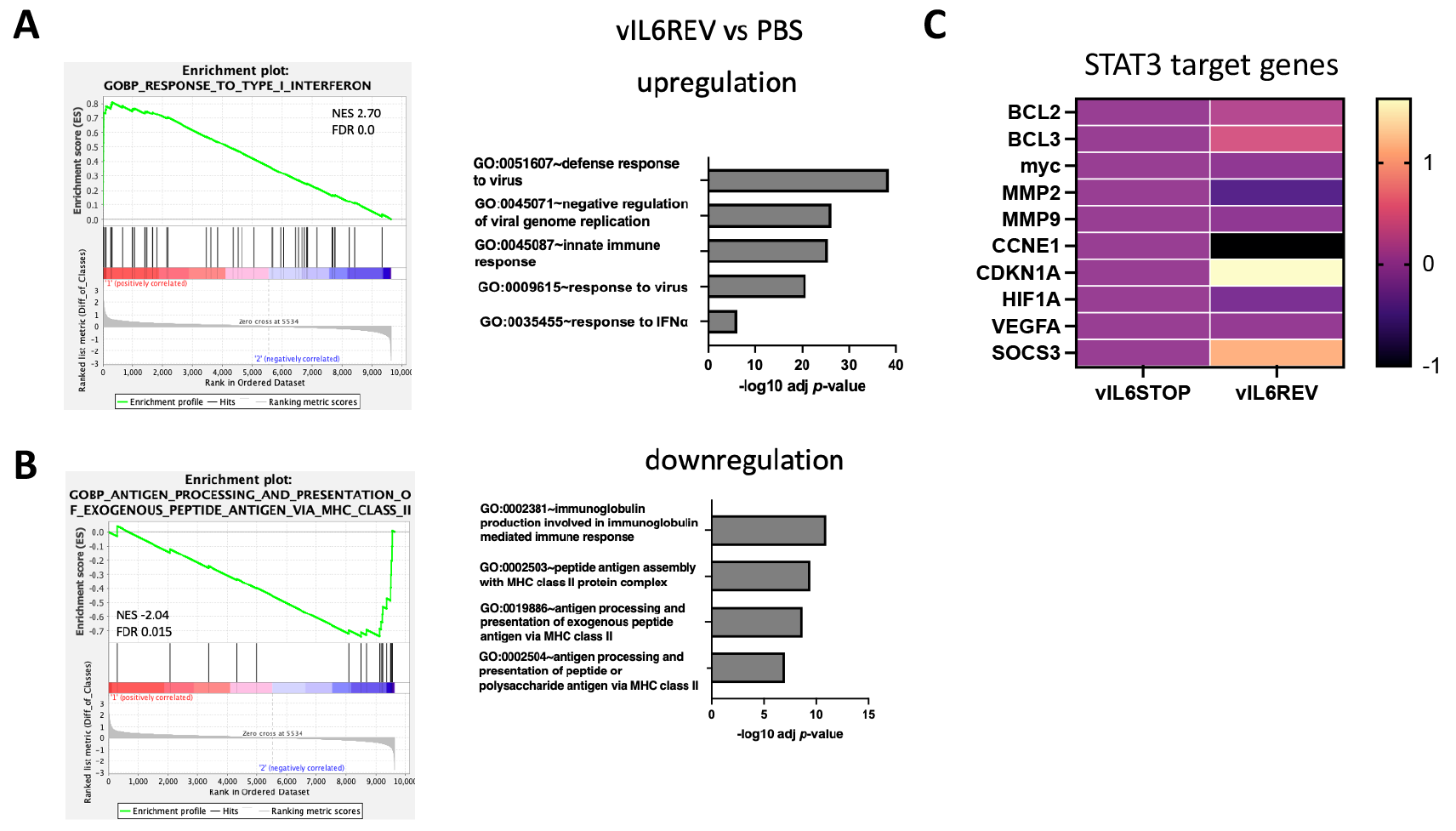

Supplement: S7 Fig — The functional enrichment analysis of upregulated genes (A) and downregulated genes (B) in vIL6REV- compared to mock-infected (PBS treated) monocytes are shown. DAVID functional enrichment analysis was performed, and the top 5 enriched pathway are shown (Right). The representative results of GSEA are shown (Left). (C) Heatmap. Heatmap of representative STAT3 downstream genes expression between vIL6STOP-infected monocytes and vIL6REV-infected monocytes. The Log2 fold change (FC) is shown when genes expression with vIL6STOP-infected monocytes is set to 1. (TIF) [file ppat.1011703.s008.tif]

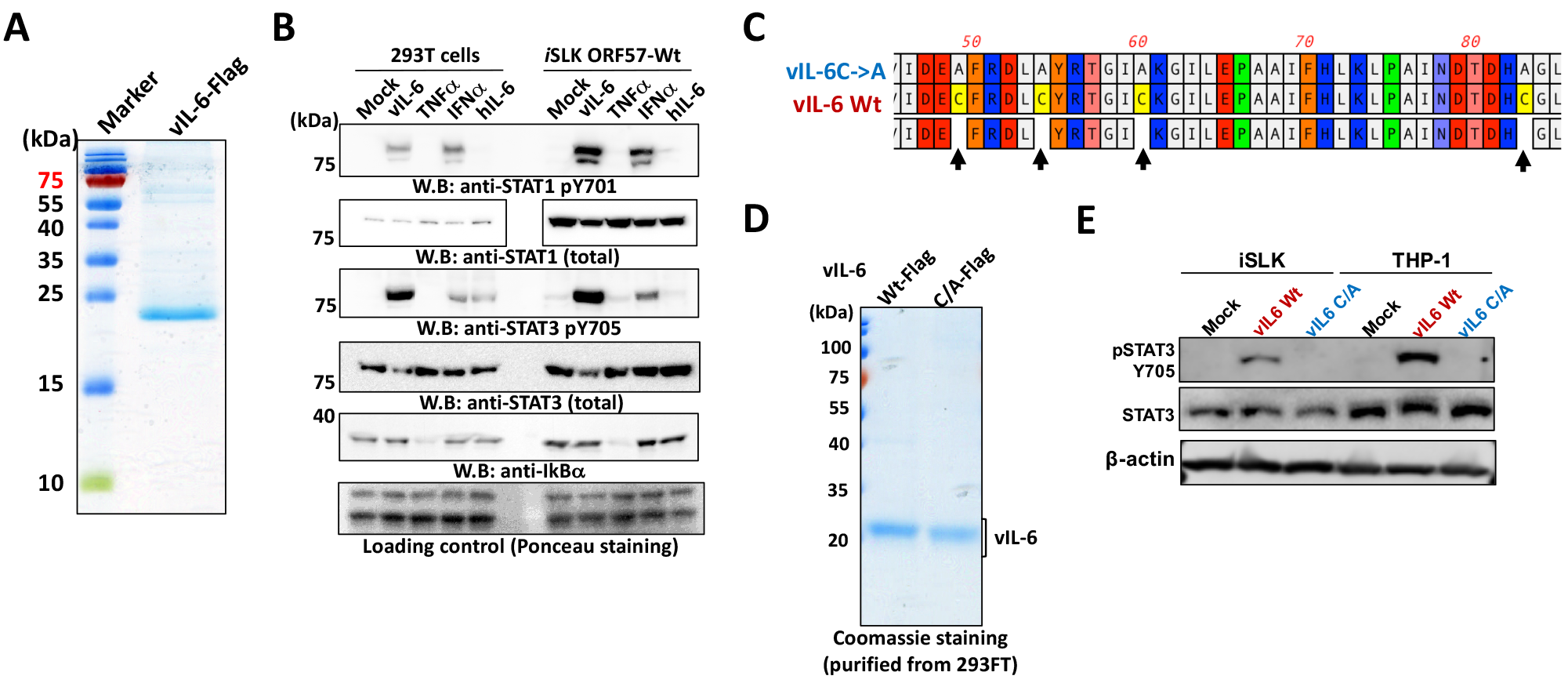

Supplement: S8 Fig — (A) Recombinant vIL-6 protein. Recombinant vIL-6-Flag protein was produced in insect cell lines, and SDS-PAGE confirmed the purity. A gel image with molecular weight markers is shown. (B) Ligand activity. 293T and iSLK ORF57-wt cell lines were stimulated with 200 ng/ml recombinant vIL-6 along with controls, TNFα, IFNα, and human (h)IL-6, or mock-treated. STAT1 and 3 activations were studied by Western blotting with specific antibodies for total STAT1, STAT1 pY701, total STAT3, STAT3 pY705, and IkBα as controls. Ponceau staining for each well are shown as loading control. (C) vIL-6 Cysteine mutant. To validate vIL-6 activity, wild type (Wt) or mutant (C->A) was generated. Arrows indicate the location of amino acid residues where mutations are introduced. (D) Coomassie staining. Wt and C/A mutant vIL-6 proteins were expressed in 293FT cell lines, and the purity of recombinant proteins was examined by SDS-PAGE. (E) Confirmation of signaling activation activity. iSLK and THP-1 cell lines were stimulated with 200 ng/ml recombinant Wt or C/A mutant vIL6, or mock-treated. STAT3 activation was studied by Western blotting with specific antibodies for total STAT3, STAT3 pY705, and b-actin as controls. Wt but not C/A mutant triggered STAT3 activation. (TIF) [file ppat.1011703.s009.tif]
